# Two‐Stage Double‐Arm Trial Optimal Design of Restricted Mean Survival Time With Sculpted Critical Region

**DOI:** 10.1002/sim.70589

**Published:** 2026-05-17

**Authors:** Jiashan He, Ruitao Lin, Yaxian Chen, K. F. Lam

**Affiliations:** ^1^ Department of Statistics The Ohio State University Columbus Ohio USA; ^2^ Department of Biostatistics The University of Texas MD Anderson Cancer Center Houston Texas USA; ^3^ Innovent Biologics Suzhou China; ^4^ School of Nursing and Health Sciences Hong Kong Metropolitan University Hong Kong China; ^5^ Department of Statistics and Actuarial Science The University of Hong Kong Hong Kong China

**Keywords:** adaptive design, non‐proportional hazards, optimal design, restricted mean survival time, two‐stage design

## Abstract

We propose an optimal phase II design for double‐arm two‐stage clinical trials using the restricted mean survival time (RMST) to measure the between‐arm difference. In the superiority test of the proposed design, the null hypothesis is rejected when the between‐arm RMST difference ${\hat{R}}_{E}-{\hat{R}}_{C}>m$, and the RMST in the experimental arm ${\hat{R}}_{E}>q$, where the critical values m and q are determined by an adaptive probability cutoff function with accumulated sample size at each stage and the asymptotic normality of RMST values. Compared to rejection rule based on ${\hat{R}}_{E}-{\hat{R}}_{C}>m$ only, the sculpted critical region requires smaller sample size based on the same type I error and power level. In the two‐stage Minimax and Optimal design, simulations demonstrate that the Sculpted RMST approach offers lower total sample size, earlier interim time (smaller interim sample size under constant accrual rate), and smaller expected sample sizes compared to the log‐rank test and simple RMST difference test. Besides, the method can be easily extended to multi‐stage sequential design based on the adaptive probability cutoff function. The global robustness of type I error deviation when the survival parameter drift from the assumption is discussed. Implementation of the proposed design on real world trial data is also provided. The R package ScuRMST for implementing the design is now available on Github.

## 1 Introduction

Within therapeutic development pipelines, phase II trials evaluate intervention efficacy following Phase I safety confirmation, thereby informing progression decisions to Phase III studies. Double‐arm trial compares the response rate or survival time between the experimental and control groups. Furthermore, two‐stage design provides an opportunity to optimize resources and enhance efficiency by early termination of the trial for futility when interim result shows the experimental group is unlikely to outperform the standard regimen.

Simon [1] proposed an optimal two‐stage single‐arm design for binary endpoints with minimal expected sample size and minimal total sample size criteria while controlling type I and type II error rates, and was extended to the two‐stage double‐arm design by Jung [2]. Denote E and C the number of successes in the experimental and Control groups, respectively. The treatment will be concluded effective when E−C>m in traditional double‐arm trials, where m is the critical value. Litwin et al. [3] designed a sculpted critical region E−C>m∩E>q, where q is a sufficiently large number. This sculpted region can efficiently reduce type I error locally near the stated null hypothesis by removing points with lower than expected numbr of successes in the control arm. Moreover, Bayesian approaches are gaining attention, such as BOP2 by Zhou et al. [4], which introduced a parameter optimization function to adjust the probability of early stopping while controlling the overall type I error. This method bridges the gap between Bayesian and frequentist designs.

With time‐to‐event endpoint, the log‐rank test [5] has been utilized to exam the equality of two survival distributions when censoring is present. Kwak and Jung [6] proposed the optimal two‐stage log‐rank test based on the asymptotic joint normality of the log‐rank statistics in the first and second stages. Royston and Parmar [7] introduced restricted mean survival time (RMST) as a new measure. Huang and Kuan [8] considered a single‐stage, double‐arm superiority test. Simulation results showed that the test based on RMST difference is more powerful than the log‐rank test in most non‐proportional hazards scenarios. Eaton et al. [9] provided a closed form power formula for a single‐stage double‐arm trial based on RMST difference. One step further, Chen et al. [10] derived a sample size formula for multi‐arm parallel single‐stage design with RMST, which integrated most studies in RMST single‐stage design. Shan [11] considered an optimal two‐stage RMST design by grid searching the critical values directly. Lu and Tian [12] introduced a group‐sequential design with RMST by the asymptotic joint normality of RMST difference between groups across different stages. The covariance formula of RMST difference was derived but Monte‐Carlo simulation is applied in practice [12]. There is still a lack of research on two‐stage design with RMST compared to single‐stage. An innovative two‐stage design based on RMST is proposed to make the trial more interpretable for clinicians. Additionally, the critical region was optimized to achieve higher power under the same type I error constraint, which in turn reduces the required sample size in the trial design.

Let ${\hat{R}}_{E}$ and ${\hat{R}}_{C}$ be estimated RMST of the experimental and control groups, respectively. We propose a sculpted region ${\hat{R}}_{E}-{\hat{R}}_{C}>m\;\cap \;{\hat{R}}_{E}>q$ in both stages to re‐allocate the overall type I error as in Litwin et al. [3]. Due to the nature of underlying asymptotic normal distribution of the RMST estimator [10], the critical values of two‐stage design cannot be enumerated like the binomial calculation for binary endpoint. So we introduce a control function of interim sample size, which is enlightened by the optimization function in BOP2 with time‐to‐event endpoint [13], to manage the probability of terminating the trial in interim and to provide equality constraint for critical value determination. In two‐stage design that terminating at interim for futility only, smaller empirical type I error is associated with higher probability of early termination under the null hypothesis. The proposed design is able to provide smaller expected sample size in the optimal design.

The paper is organized as follows, Section 2 will introduce single‐stage simple RMST difference test. Details of sculpted regions for a two‐stage design will be illustrated in Section 3. The control function will be defined in Section 3.3 and the critical value calculation by Monte Carlo will be presented in Section 3.4. The minimax and optimal designs searching procedure is introduced in Section 3.5 to meet the requirements in actual clinical trials. In Section 4, we conducted a series of comparative experiments in different scenarios among the proposed sculpted RMST, simple RMST and log‐rank test. The visualized critical regions and robustness under different hypothesis parameters will be discussed in Sections 4.4 and 4.5 respectively to further understand the nature of proposed method. Concluding remarks and discussion are provided in Section 5.

## 2 Double‐Arm Trial With Restricted Mean Survival Time

Let T be the survival time with survival function S(t). The restricted mean survival time [7] (RMST) is defined as $R\left(\tau \right)=E\left(\min\left(T,\tau \right)\right)=\int_{0}^{\tau }S\left(t\right)\mathrm{dt}$, where τ is the restricted upper bound of T. R(τ) can be consistently estimated by the area under $\hat{S}\left(t\right)$, the Kaplan–Meier estimates [14], as

$$\hat{R}\left(\tau \right)=\int_{0}^{\tau }\hat{S}\left(t\right)\mathrm{dt}=\sum_{j=0}^{D}\left(t_{j+1}-t_{j}\right)\hat{S}\left(t_{j}\right),$$

where $t_{i}$ is the distinct event time before restricted cut‐off time τ satisfying $0<t_{1}<t_{2}<\ldots <t_{D}<t_{D+1}=\tau$. The RMST estimator is asymptotically valid for t<τ. All RMST values in this paper are estimated by R package ‘survRM2’ [15] ‘rmst2’ function.

RMST can be used as a measure for making decisions in clinical trials. In this study, we will discuss double‐arm 1:1 randomization trials, where patients are randomly allocated to either the experimental or the control group. Since the RMST can be intuitively interpreted as the life expectancy before time τ, large value of RMST in the experimental group often indicates pronounced treatment effect. So a superiority trial based on a one‐sided hypothesis testing can be considered with

$$H_{0}:R_{E}\left(\tau \right)=R_{C}\left(\tau \right)\;\text{against}\;H_{1}:R_{E}\left(\tau \right)>R_{C}\left(\tau \right),$$

where E and C stand for the experimental and control groups, respectively. The survival functions in two arms are denoted by $S_{E}\left(t\right)\;\text{and}\;S_{C}\left(t\right)$. The estimator of the RMST difference between the two arms and its variance before a common cut‐off time τ can be estimated by 

(1)

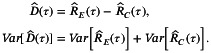




Due to the asymptotic standard normality of $\hat{D}\left(\tau \right)$ under the null hypothesis [14], the 100(1−α)% confidence interval for D(τ) is $\hat{D}\left(\tau \right)\pm z_{1-\alpha /2}\sqrt{\mathrm{Var}\left[\hat{D}\left(\tau \right)\right]}$, where $z_{\alpha }$ is the upper 100α% quantile of the standard normal distribution. The traditional one‐sided (greater) hypothesis test with the type I error rate α can be denoted as rejecting $H_{0}$ and declaring the drug or treatment effective when

(2) $$\hat{D}\left(\tau \right)={\hat{R}}_{E}\left(\tau \right)-{\hat{R}}_{C}\left(\tau \right)>z_{1-\alpha }\sqrt{\mathrm{Var}\left[\hat{D}\left(\tau \right)\right]}.$$

## 3 Two‐Stage Trial Design With Sculpted Critical Region

**FIGURE 1:**
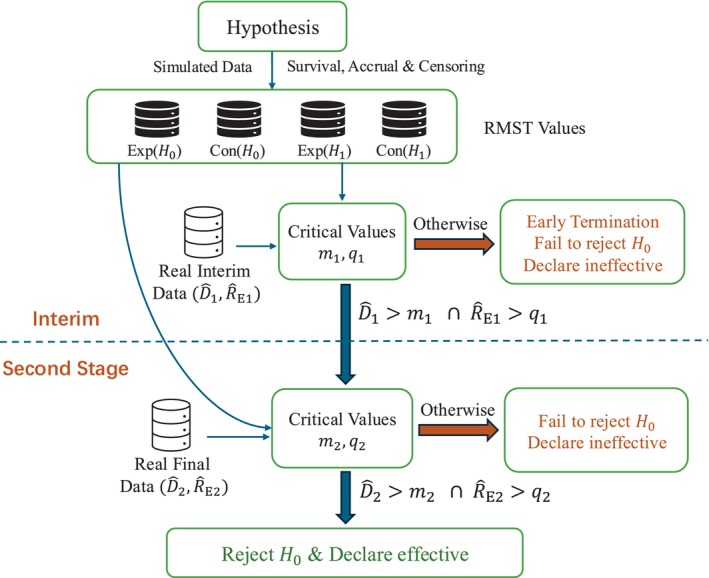
Flow chart of the trial. $\hat{D}\;\text{and}\;{\hat{R}}_{E}$ are the estimated RMST difference between two groups and RMST of experiment group respectively. At interim, ${\hat{R}}_{E1}\left(\tau_{1}\right)$ and ${\hat{D}}_{1}\left(\tau_{1}\right)={\hat{R}}_{E1}\left(\tau_{1}\right)-{\hat{R}}_{C1}\left(\tau_{1}\right)$ are estimated. At final stage, ${\hat{R}}_{E2}\left(\tau_{2}\right)$ and ${\hat{D}}_{2}\left(\tau_{2}\right)={\hat{R}}_{E2}\left(\tau_{2}\right)-{\hat{R}}_{C2}\left(\tau_{2}\right)$ are estimated. The treatment can only be declared effective at the final stage.

The flow chart of the proposed design is shown in Figure 1 for better comprehension. The simulated RMST values under a certain pair of hypotheses will be used to calculate the critical values in two stages. During the interim, the trial will be terminated and declared ineffective if either ${\hat{D}}_{1}$ or ${\hat{R}}_{E1}$ in the real clinical data is smaller than its corresponding critical value. Otherwise, patients and data from the interim period will be retained and continuously observed in the second stage, where the same rejection method is applied with different critical values. In conclusion, the null hypothesis $H_{0}$ can only be rejected if the values $\hat{D}$ and ${\hat{R}}_{E}$ in both stages are greater than their corresponding critical values.

### 3.1 Critical Region Sculpting

The critical region construction in Equation (2) is intuitive and easy to implement in clinical trials. Under the stated null hypothesis $H_{0}:R_{E}\left(\tau \right)=R_{C}\left(\tau \right)$, random variation may produce samples in which ${\hat{R}}_{C}\left(\tau \right)$ is unusually small. In such cases, the RMST difference $\hat{D}\left(\tau \right)={\hat{R}}_{E}\left(\tau \right)-{\hat{R}}_{C}\left(\tau \right)$ may exceed the rejection boundary even when ${\hat{R}}_{E}\left(\tau \right)$ itself is not large in absolute terms. Although this corresponds to a valid rejection rule for testing $H_{0}$, it may be clinically unconvincing because the experimental arm does not demonstrate a sufficiently large RMST. Motivated by this consideration, we introduce the sculpted critical region to reduce the chance of such control‐driven rejections while maintaining the overall type I error. The proposed rejection region is denoted as rejecting $H_{0}$ when

$$\hat{D}\left(\tau \right)>m\;\cap \;{\hat{R}}_{E}\left(\tau \right)>q,$$

where m and q are to be determined. This sculpted critical region can hopefully reduce the type I error rate of the trial compared with the original one under the same setting and can be intuitively applied to each stage of the trial.

### 3.2 Interim Analysis

In a two‐stage phase II trial, an interim analysis will be performed during patient recruitment to assess whether the trial should be terminated early for efficacy, futility, or both. After determining the overall sample size N, we plan to conduct the interim analysis when the number of recruited patients reaches $\tilde{N}$, where $\tilde{N}$ denotes the total interim sample size of two groups. The sculpted critical region is naturally suitable for early stopping for futility only, which means that the trial should be terminated when either ${\hat{D}}_{1}\left(\tau_{1}\right)\leq m_{1}$ or ${\hat{R}}_{E1}\left(\tau_{1}\right)\leq q_{1}$. The subscript 1 under each variable represents the corresponding value in the interim period. The probability of early termination (PET) during the interim period is defined as

(3) $${\mathrm{PET}}_{h}=P\left({\hat{D}}_{1}\left(\tau_{1}\right)\leq m_{1}\;\cup \;{\hat{R}}_{E1}\left(\tau_{1}\right)\leq q_{1}\;|\;H_{h}\right),$$

where h∈{0,1} representing the null and alternative hypotheses, respectively. The treatment will be declared ineffective if the trial is terminated early. Although it is theoretically possible to terminate for both superiority and futility, it requires calculating four critical values at each stage, which adds considerable complexity. Thus, this research will focus on early termination solely for futility.

### 3.3 Second Stage Decision

One main challenge of the two‐stage design is to build a bridge between interim and final analyses. For non‐adaptive trial design, both the early stopping rule and the final critical value are typically determined when the protocol is established. The $H_{0}$ will be rejected when

$${\hat{D}}_{1}\left(\tau_{1}\right)>m_{1}\;\cap \;{\hat{R}}_{E1}\left(\tau_{1}\right)>q_{1}\;\cap \;{\hat{D}}_{2}\left(\tau_{2}\right)>m_{2}\;\cap \;{\hat{R}}_{E2}\left(\tau_{2}\right)>q_{2}.$$

${\hat{D}}_{i}\left(\tau_{i}\right)$ are the estimated RMST difference defined in Equation (1) with the subscript representing the interim and final stage. ${\hat{R}}_{\mathrm{Ei}}\left(\tau_{i}\right)$ is the RMST of the experimental arm in stage i. The cut‐off point $\tau_{1}$ can be chosen as the minimum of the maximum observed follow‐up times (including censored times) in the two arms at the interim analysis so that the interim RMSTs are computed on a common time horizon and use the widest time window supported by the interim data in both arms. This choice is consistent with the results of Tian et al. [16], which justify inference for RMST with a data‐dependent truncation time up to the largest observed follow‐up time under mild conditions on the censoring distribution. $\tau_{2}$ for the final stage can be specified during the design phase to reflect a clinically relevant time to enhance the interpretability of final decision. In stage i, the additional constraint imposed by ${\hat{R}}_{\mathrm{Ei}}$ complicates the direct calculation of the critical values $m_{i}$ and $q_{i}$. This is because there are infinitely many possible solutions for $\left(m_{i},q_{i}\right)$ that satisfy the probability condition $P\left({\hat{D}}_{i}>m_{i}\cap {\hat{R}}_{\mathrm{Ei}}>q_{i}\right)=p_{0}$ when $\left({\hat{D}}_{i},{\hat{R}}_{\mathrm{Ei}}\right)$ follows an asymptotic bivariate normal distribution according to the asymptotic normality of the RMST estimator [7]. Besides, the link between RMST values at interim and at final stage need to be determined. Therefore, additional restrictions are required to compute the values $\left(m_{1},q_{1},m_{2},q_{2}\right)$.

The stringent sculpted critical region aims to preserve the overall Type I error rate, but it may be overly strict during the interim study when the sample size is insufficient. This may hinder progression to the second cohort. In a two‐stage phase II trial, the early stopping threshold for the statistics at interim should be relatively lenient when accrual sample size $\tilde{N}$ is small. It allows for the collection of more evidence in the second stage to assess treatment effectiveness. In the Bayesian study BOP2 [13], the proposed function $C\left(\tilde{N}\right)=1-\lambda {\left(\tilde{N}/N\right)}^{\gamma }$ with flexible parameters (λ,γ) was used to control early stopping probability when the sample size accumulates to $\tilde{N}$. The trial is designed to be terminated when $P\left({\tilde{\mu }}_{E}\leq {\tilde{\mu }}_{C}\;|\;D_{\tilde{N}}\right)>C\left(\tilde{N}\right)$, where $\tilde{\mu }\;|\;D_{\tilde{N}}$ is the posterior median progression‐free survival with data up to $\tilde{N}$ patients in BOP2 [13]. Meanwhile in our study, another control function $f\left(\tilde{N}\right)$ is introduced to assign a loosened critical value at interim for small $\tilde{N}/N$ with

$$f\left(\tilde{N}\right)=\exp\left(-\gamma \cdot \frac{\tilde{N}}{N}\right),\;\gamma >0.$$

$f\left(\tilde{N}\right)$ is a monotonically decreasing function about interim sample size $\tilde{N}$, where the total sample size N is fixed as a constant. Since $0<\tilde{N}/N<1$, $f\left(\tilde{N}\right)\in \left(0,1\right)$. Compared to $C\left(\tilde{N}\right)=1-\lambda {\left(\tilde{N}/N\right)}^{\gamma }$, our function f requires only a single parameter to control the probability threshold, which has been proven sufficient through extensive simulation. Based on this control function, we propose a specific constraint for each stage under the alternative hypothesis $H_{1}$ to calculate the critical values as follows:

(4) $$\begin{array}{l} \;P\left({\hat{R}}_{E1}\left(\tau_{1}\right)>q_{1}\;|\;{\hat{D}}_{1}\left(\tau_{1}\right)>m_{1}\right)=f\left(\tilde{N}\right);\\ P\left({\hat{R}}_{E2}\left(\tau_{2}\right)>q_{2}\;|\;{\hat{D}}_{2}\left(\tau_{2}\right)>m_{2}\right)=f\left(N\right). \end{array}$$

When $\left(\gamma ,\tilde{N},N\right)$ are fixed, $\left(m_{1},\;q_{1},\;m_{2},\;q_{2}\right)$ can be solved by a procedure described in Section 3.4. At interim stage, grid search suitable ${\hat{D}}_{1}\left(\tau_{1}\right)$ values in simulated survival data for its critical value $m_{1}$. For each fixed $m_{1}$, the corresponding critical value $q_{1}$ can be calculated based on the probability threshold. So does $\left(m_{2},q_{2}\right)$. The motivation of critical value sculpting is to assign a proper boundary for RMST value in one arm to mitigate the large between‐group difference required in the rejection region of two‐sample test. $f\left(N_{i}\right)$ acts as a bridge between interim and final stages. It specifies the conditional probability threshold that determines how stringent the additional sculpting requirement ${\hat{R}}_{\mathrm{Ei}}\left(\tau_{i}\right)>q_{i}$ is, given that ${\hat{D}}_{i}\left(\tau_{i}\right)>m_{i}$. Because $f\left(\tilde{N}\right)>f\left(N\right)$, the interim analysis applies a less stringent conditional probability threshold than the final analysis. Note that this does not guarantee $q_{1}<q_{2}$ since the underlying conditions differ between stages. The tuning parameter γ controls the rate at which the design transitions from lenient sculpting at interim to stricter sculpting at the final analysis.

There are two more natural restrictions, which are type I error rate and power constraint, apart from these two designed restrictions. The type I error rate α and power of this test are defined as 

(5)

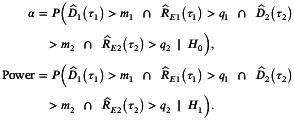




The parameter γ in $f\left(\tilde{N}\right)$ provides flexibility to search for more critical values combinations that better fits the RMST distribution under both hypotheses. Besides, this function also offers adaptivity by automatically adjusting the strictness of critical values based on the sample size at each stage. It can be easily adapted to multi‐stage design by appending an additional restriction equation at each stage.

### 3.4 Critical Value Calculation

#### 3.4.1 Notations

Let $T_{\mathrm{ak}}$ be the survival time of patient k in arm a, where k∈{1,2,…N/2},a=EorC for experimental and control groups, respectively. $T_{\mathrm{ak}}$ follows a certain distribution with survival function $S_{a}$. $C_{\mathrm{ak}}$ is the censoring time after the patients' entry following the censoring distribution $U_{\mathrm{cen}}$. $E_{\mathrm{ak}}$ is the entry time of patient following the accrual distribution $U_{\mathrm{acc}}$. The explicit expressions of $S_{E},\;S_{c}\;,\;U_{\mathrm{cen}}\;\text{and}\;U_{\mathrm{acc}}$ are required for the following estimation. $\delta_{\mathrm{ak}}$ is the event indicator taking value 1 when the event occurs for patient k and 0 otherwise. If the interim period is set at the calendar time T, the observed time ${\tilde{O}}_{\mathrm{ak}}$ in interim is 


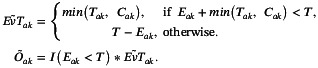




The observed data at interim is $\left({\tilde{\delta }}_{a},{\tilde{O}}_{a}\right)$. We only discuss conducting interim analysis once during the patient accrual period, which can possibly reduce the total sample size of the trial. The ongoing patient recruitment and observation ends at the final analysis when the observed survival data is denoted as $\left(\delta_{a},O_{a}\right)$. Please note that the cutoff time τ of RMST will be omitted for tidy formulae demonstration in the rest of this section.

The goal is to solve for the critical values $\left(m_{1},q_{1},m_{2},q_{2}\right)$ given $\left(\gamma ,\tilde{N},N\right)$ in Equation (4). The marginal distribution of ${\hat{D}}_{i}$ and the joint distribution of $\left({\hat{D}}_{i},{\hat{R}}_{\mathrm{Ei}}\right)$ are crucial for the calculation. Lu and Tian [12] derived an explicit form of variance–covariance matrix estimator of sequential between group RMST differences according to the asymptotic joint normality of $\left({\hat{D}}_{1},{\hat{D}}_{2}\ldots \;{\hat{D}}_{i}\right)$. Monte‐Carlo method is adopted while implementing the estimation in group‐sequential trial design due to the complexity and time‐consuming nature of numerical integration. Similarly, Monte‐Carlo is also applicable in our case and can be easily conducted according to the marginal and joint normality of RMST estimators within each stage.

#### 3.4.2 Two‐Stage Design Under α Constraint

We begin by introducing the process for determining the critical values that maximize the overall power while preserving a specified type I error rate. First, the total sample size N and interim sample size $\tilde{N}$ must be prespecified. Based on the restrictions we defined at Equation (4), for any given $\left(m_{i},\gamma ,\tilde{N},N\right)$, the critical value $q_{i}$ is the quantile of the distribution of ${\hat{R}}_{\mathrm{Ei}}$ conditioned on ${\hat{D}}_{i}>m_{i}$. We start with the distribution of ${\hat{R}}_{\mathrm{Ei}}\;|\;{\hat{D}}_{i}=m_{i}$. Due to the normality of RMST estimators [7], $\left({\hat{R}}_{\mathrm{Ei}},{\hat{D}}_{i}\right)$ follows a bivariate normal distribution asymptotically. We assume the marginal distribution is ${\hat{R}}_{\mathrm{Ei}}∼N\left(\mu_{E_{i}},\sigma_{E_{i}}^{2}\right)$ and ${\hat{D}}_{i}∼N\left(\mu_{D_{i}},\sigma_{D_{i}}^{2}\right)$. $\rho_{i}$ denotes the correlation between ${\hat{D}}_{i}\left(\tau_{i}\right)$ and ${\hat{R}}_{\mathrm{Ei}}\left(\tau_{i}\right)$ under the bivariate normal approximation. The conditional distribution of ${\hat{R}}_{\mathrm{Ei}}\;|\;{\hat{D}}_{i}$ is also normal asymptotically, which is:

(6) $${\hat{R}}_{\mathrm{Ei}}|{\hat{D}}_{i}=x\;\dot{∼}N\left(\mu_{\mathrm{Ei}}+\rho_{i}\frac{\sigma_{\mathrm{Ei}}}{\sigma_{\mathrm{Di}}}\left(x-\mu_{\mathrm{Di}}\right),\;\left(1-\rho_{i}^{2}\right)\sigma_{\mathrm{Ei}}^{2}\right),\;x\in \left(-\infty ,\infty \right).$$

We set $x=m_{i}$ for notational consistency with Equation (5). The mean and variance of ${\hat{D}}_{i}\;|\;{\hat{D}}_{i}>m_{i}$ is:

(7) $$E\left[{\hat{R}}_{\mathrm{Ei}}|{\hat{D}}_{i}>m_{i}\right]=\mu_{E_{i}}+\rho_{i}\sigma_{E_{i}}\frac{\phi \left(\theta \right)}{1-\Phi \left(\theta \right)}.$$

(8) $$\mathrm{Var}\left({\hat{R}}_{\mathrm{Ei}}|{\hat{D}}_{i}>m_{i}\right)=\left(1-\rho_{i}^{2}\right)\sigma_{E_{i}}^{2}+{\left(\rho_{i}\frac{\sigma_{E_{i}}}{\sigma_{D_{i}}}\right)}^{2}\cdot \sigma_{D_{i}}^{2}\left[1-\frac{\theta \cdot \phi \left(\theta \right)}{1-\Phi \left(\theta \right)}-{\left(\frac{\phi \left(\theta \right)}{1-\Phi \left(\theta \right)}\right)}^{2}\right]$$

where:

$$\theta =\frac{m_{i}-\mu_{D_{i}}}{\sigma_{D_{i}}},$$

ϕ(θ) is the standard normal probability density function evaluated at θ, and Φ(θ) is the standard normal cumulative distribution function evaluated at θ. The derivation can be found in the Supporting Information: Appendix.

Thus, given the value of $m_{i}$, the mean and variance of ${\hat{R}}_{\mathrm{Ei}}|{\hat{D}}_{i}>m_{i}$ at stage i can be estimated by Equations (7) and (8), respectively. Then a large number, say B = 10 000 groups of survival data are generated for arm a under $H_{0}$ and $H_{1}$. They are denoted as ${\left({\tilde{\delta }}_{a},{\tilde{O}}_{a},\delta_{a},O_{a}\;|\;H_{h}\right)}_{j},\text{where}\;j\in \left\{1,2,\ldots B\right\}$, a=EorC for two arms under hypothesis h=0or1. Values observed at interim are denoted by characters with a tilde, while those observed at the final stage are denoted by characters without. It means that totally 4B groups of data are generated under a pair of hypotheses $\left(H_{0},H_{1}\right)$. The RMST of both arms and the between‐groups difference can be estimated, which is denoted as ${\left({\hat{D}}_{1},{\hat{R}}_{E1},{\hat{D}}_{2},{\hat{R}}_{E2}\;|\;H_{h}\right)}_{j}$, where subscripts 1 and 2 represent the stage. We first consider ${\left({\hat{D}}_{1},{\hat{R}}_{E1}\;|\;H_{1}\right)}_{j}$. Due to the asymptotic normality of RMST values under a certain specified form of $H_{1}$ [7],

$$\left(\begin{array}{c} {\hat{D}}_{1}-D_{1}\\ {\hat{R}}_{E1}-R_{E1} \end{array}\right)\dot{∼}\;𝒩\left(0,\sum_{1}\right),$$

where $D_{1}$ and $R_{E1}$ are the theoretical integration of RMST defined in Section 2 by the form of assumed survival function in $H_{1}$. $\sum_{1}$ can be estimated by Monte‐Carlo approach with the process being shown below

(9) $$I_{j}=\left(\begin{array}{c} {\hat{D}}_{1j}-{\bar{D}}_{1}\\ {\hat{R}}_{E1j}-{\bar{R}}_{E1} \end{array}\right),\;{\bar{D}}_{1}=\frac{1}{B}\sum_{j=1}^{B}{\hat{D}}_{1j},\;{\bar{R}}_{E1}=\frac{1}{B}\sum_{j=1}^{B}{\hat{R}}_{E1j},\;{\hat{\sum }}_{1}=\frac{1}{B}\sum_{j=1}^{B}I_{j}I_{j}^{⊤}.$$

$D_{1}$ is subtracted from ${\hat{D}}_{1}$ in the original expression. But in practice, $D_{1}$ is replaced by the mean value of Monte‐Carlo simulated data ${\bar{D}}_{1}$ in the estimator. Same for ${\bar{R}}_{E1}$. The asymptotic bivariate normality [12] of $\left({\hat{D}}_{1},{\hat{D}}_{2}\right)$ is not applied in our Monte‐Carlo simulation. Instead, the control function f in Equation (4) can be regarded as a bridge between interim and the second stage. After that, the estimation of ${\hat{D}}_{2}$ and ${\hat{R}}_{E2}$ at the final stage are the same as the procedure stated above and estimated variance–covariance matrix of second is denoted as ${\hat{\sum }}_{2}$. We now obtain the mean values ${\bar{D}}_{1}$, ${\bar{R}}_{E1}$, ${\bar{D}}_{2}$, ${\bar{R}}_{E2}$ and the covariance matrices ${\hat{\sum }}_{1}$, ${\hat{\sum }}_{2}$ for the two stages. From these, the estimated mean and variance of $\left({\hat{R}}_{\mathrm{Ei}},{\hat{D}}_{i}\right)$ at stage i used in Equation (6) can also be collected. There is no guarantee that the conditional distribution of ${\hat{R}}_{\mathrm{Ei}}|{\hat{D}}_{i}>m_{i}$ is also asymptotic truncated normal. While it is feasible to sample from the distribution of ${\hat{R}}_{\mathrm{Ei}}|{\hat{D}}_{i}>m_{i}$, this requires sampling for every $m_{i}$ value, which is extremely time consuming and computationally burdensome. For ease of calculation, we apply a normal approximation to calculate the critical value $q_{i}$ given $m_{i}$. Experiments in Section 4.2 demonstrates the efficacy of this approach. It is worth noting that only the data simulated under the alternative hypothesis are used for the estimation [12]. Data under the null hypothesis will help control the empirical type I error in the following searching procedure. Consequently, the critical values in Equation (4) achieving a certain power level under α constraint can be determined by the following steps:

 

Two‐stage design given total and interim sample size

1. Given ($\alpha_{0},\beta_{0},S_{E},S_{C},U_{\mathrm{acc}},U_{\mathrm{cen}},\tilde{N},N$). Generate the survival data ${\left({\tilde{\delta }}_{i},{\tilde{O}}_{i},\delta_{i},O_{i}\;|\;H_{h}\right)}_{j},\;j\in \left\{1,\ldots B\right\}$ for the two arms i=EandC under hypothesis $H_{0}$ and $H_{1}$, respectively, with a total of 4B samples of data.
2. Estimate RMST values ${\left({\hat{D}}_{1},{\hat{R}}_{E1},{\hat{D}}_{2},{\hat{R}}_{E2}\;|\;H_{h}\right)}_{j},\;j\in \left\{1,\ldots B\right\}$ based on the simulated data. Totally 2B groups of RMST data.
3. Denote RMST_H0 = $\left({\hat{D}}_{1},{\hat{R}}_{E1},{\hat{D}}_{2},{\hat{R}}_{E2}\;|\;H_{0}\right)$, RMST_H1 = $\left({\hat{D}}_{1},{\hat{R}}_{E1},{\hat{D}}_{2},{\hat{R}}_{E2}\;|\;H_{1}\right)$ each containing B groups of data respectively. Obtain $\left({\bar{D}}_{1},{\bar{R}}_{E1},{\bar{D}}_{2},{\bar{R}}_{E2},{\hat{\sum }}_{1},{\hat{\sum }}_{2}\;|\;H_{1}\right)$ by Monte‐Carlo in Equation (9).
4. Grid search parameters $\left(\gamma ,m_{1},m_{2}\right)$ in Equation (4).

       $\forall \left(\gamma ,m_{1},m_{2}\right)$ and given $\left(\tilde{N},N\right)$:

    Estimate $\left(q_{1},q_{2}\right)$ as the quantile of ${\hat{R}}_{E1}|{\hat{D}}_{1}>m_{1}$ and ${\hat{R}}_{E2}|{\hat{D}}_{2}>m_{2}$ using Equation (7) and (8).

5 Plug $\left(m_{1},\;q_{1},\;m_{2},\;q_{2}\right)$ in RMST_H0 for the empirical type I error $\alpha_{E}$ and RMST_H1 for empirical power ${\text{power}}_{E}$ by Equation (5).
6 Collect all $\left(m_{1},\;q_{1},\;m_{2},\;q_{2}\right)$ that fulfill $\alpha_{E}\leq \alpha_{0}$. Select one with closest ${\text{power}}_{E}$ to 1‐$\beta_{0}$ as critical values.

The fundamental logic of this procedure is to identify the most effective decision boundaries for ${\hat{D}}_{i}$ and ${\hat{R}}_{\mathrm{Ei}}$ using the empirical distribution of four groups of simulated survival data. The critical values $\left(m_{1},\;q_{1},\;m_{2},\;q_{2}\right)$ satisfying type I error and power requirement will be determined for any given $\left(\tilde{N},N\right)$ using the asymptotic normality of $\left({\hat{D}}_{i},{\hat{R}}_{\mathrm{Ei}}\right)$. Searching for the parameter γ can help to balance the stopping probabilities of two stages that best fit the simulated data. When implementing the search grid with $\left(\gamma ,m_{1},m_{2}\right)$ in step 4, we only assign γ as the loop variable. For each γ, we span all combinations of feasible $\left(m_{1},m_{2}\right)$ in a matrix. Using array‐wise functions to calculate $\left(q_{1},q_{2}\right)$ based on the estimated mean and variance of ${\hat{R}}_{\mathrm{Ei}}|{\hat{D}}_{i}>m_{i}$ can make the grid search more efficient.

### 3.5 Minimax and Optimal Design

The calculation of critical values for the two‐stage design with prespecified total sample size N and interim sample size $\tilde{N}$ is described in the previous subsection. However, clinicians often need to determine the minimal sample size required to achieve a specific power level while adhering to a type I error rate constraint in real‐world trial design. To address this need, we propose a searching method to obtain the minimal sample size and corresponding critical values for practical use.

Simon [1] proposed two optimal criteria. The first criterion aims to minimize the expected sample size under the null hypothesis, denoted as ${\mathrm{EN}}_{0}$, and is referred to as the optimal design. The second criterion, known as the minimax design, seeks to minimize the maximum total sample size N. We also use the average expected sample size [6] under both hypotheses as a measurement with the definition 

(10)

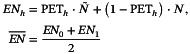


where h∈{0,1} indicating the hypothesis. The PET defined in Equation (3) and $\bar{\mathrm{EN}}$ above are empirical values based on Monte‐Carlo simulation. The optimal design can be found by the following steps:

 

Two‐stage Optimal Design Minimizing ${\mathrm{EN}}_{0}$ or N

1. Specify $\left(\alpha_{0},\;\beta_{0}\right)$ and survival settings $\left(S_{E},S_{C},U_{\mathrm{acc}},U_{\mathrm{cen}}\right)$.
2. Find the minimum sample size $n_{0}$ of a single‐stage one‐sided RMST test defined in Equation (2) fulfilling $\left(\alpha_{0},\;\beta_{0}\right)$ constraint.
3. Grid search inteirm and total sample size $\left(\tilde{N},N\right)$ by a double loop:

        $\forall N_{i}\geq 0.9n_{0}:$

                 $\forall {\tilde{N}}_{j}<N_{i}:$

- Calculate the critical values, empirical $\alpha_{E}$ and ${\text{power}}_{E}$of by step 1–5 of the procedure in Section 3.4 with $\left({\tilde{N}}_{j},N_{i}\right)$. All designs with $\left(\alpha_{E}\leq \alpha_{0}\;\cap \;{\text{power}}_{E}\geq 1-\beta_{0}\right)$will be stored.
- Calculate ${\mathrm{EN}}_{0},{\mathrm{EN}}_{1},\bar{\mathrm{EN}}$ for all stored designs in a. and save the settings $(m_{1},q_{1},m_{2},q_{2},\tilde{N},N,{\mathrm{EN}}_{0},{\mathrm{EN}}_{1}$, $\bar{\mathrm{EN}})$. If none of the designs in a. meets $\left(\alpha_{0},\beta_{0}\right)$ constraint, then skip to next $\left({\tilde{N}}_{j+1},N_{i}\right).$

       Repeat a. and b. with $N_{i}+1$

4 Among all $(m_{1}$, $q_{1}$, $m_{2}$, $q_{2}$, $\tilde{N}$, N, ${\mathrm{EN}}_{0}$, ${\mathrm{EN}}_{1}$, $\bar{\mathrm{EN}})$, Minimax design is the one with minimum N and Optimal design is that with minimum ${\mathrm{EN}}_{0}$.

In step 2, $n_{0}$ works as the reference initial sample size of the searching steps. Chen et al. [10] provided a sample size formula for multi‐arm single‐stage RMST test based on global $\chi^{2}$ test, which could be a reference of $n_{0}$. In addition, $n_{0}$ can also be empirically determined by conducting a series of single‐stage RMST tests on simulated data with increasing sample size. $n_{0}$ is the minimum sample size that meets $\left(\alpha_{0},{\text{power}}_{0}\right)$ constraint. An analogous optimal design searching procedure proposed by Kwak and Jung [6] for two‐stage log‐rank test starts from n−3, where n is the corresponding single‐stage minimum sample size with log‐rank test. Furthermore, the initial value $n_{0}$ will only affect the efficiency of minimax and optimal designs searching but not the precision. If the set of designs saved in step 3 with $N=0.9n_{0}$ is not empty, it is possible that a smaller expected sample size ${\mathrm{EN}}_{0}$ could be found with a smaller N. Therefore, the search procedure can continue from $N=0.9n_{0}-2$ and proceed by descending N until no suitable design can be found.

## 4 Comparative Experiments

### 4.1 Two‐Stage Log‐Rank Test

Kwak and Jung [6] provided an optimal two‐stage double‐arm design based on log‐rank test. The critical region of early termination for futility only in that paper [6] is $\left\{W_{1}/\sigma_{1}>c_{l}\cap W/\sigma >c\right\}$, where $W_{1}/\sigma_{1},\;W/\sigma$ are the log‐rank statistic of first and second stages, respectively. By the independent increment of the log‐rank test statistic, the correlation coefficient between $W_{1}$ and W is $\sqrt{\mathrm{var}\left(W_{1}\right)/\mathrm{var}\left(W\right)}$ under $H_{0}$ and $H_{1}$. Then they derived the asymptotic results of $E\left(W_{1}\right)$, E(W) and the convergence of variance estimators ${\hat{\sigma }}_{1}$ and $\hat{\sigma }$ under $H_{0}$ and $H_{1}$. The asymptotic distribution of $\left(W_{1}/\sigma_{1},W/\sigma \right)$ under null hypothesis is 


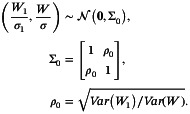




The estimation of $\rho_{0}$ under $H_{0}$ is free of proportional hazards assumption. So by the two‐stage type I error rate constraint

(11) $$P\left(\frac{W_{1}}{\sigma_{1}}>c_{l}\cap \frac{W}{\sigma }>c\;|\;H_{0}\right)=\alpha_{0},$$

for any given value of $c_{l}$, the corresponding c can be obtained by solving the integration of bivariate normal density in Equation (11). While calculating the power under $H_{1}$, the derived explicit form of variance estimators ${\hat{\sigma }}_{1}$ and $\hat{\sigma }$ in Kwak and Jung [6] is based on uniform accrual and exponential survival models, which follows the proportional hazards assumption. In order to compare the two‐stage log‐rank test with RMST methods under non‐proportional hazards assumption, we grid search for $\left(c_{l},c\right)$ in a reasonable range. The searching range can be determined by the estimated value of $\left(W_{1}/\sigma_{1},W/\sigma \right)$ in simulated data. Calculate the type I error by normal integration and collect all $\left(c_{l},c\right)$ satisfying type I error rate constraint in Equation (11). Then we estimate the empirical power of each pair of $\left(c_{l},c\right)$ using data simulated under $H_{1}$. Collect all designs fulfilling a certain type I error rate and power constraint. The optimal design search described in Section 3.5 is also applied to the two‐stage log‐rank test in the comparison of the following experiments. Table 1 will show that the performance of log‐rank test by grid searching $\left(c_{l},c\right)$ is close to the theoretical result proposed in Kwak and Jung [6] under the proportional hazards assumption.

**TABLE 1:** Minimax and optimal two‐stage designs under PH compared with the log‐rank test of Kwak and Jung [6].

α	Power	Δ	Method	${\mathrm{PET}}_{0}$	${\mathrm{PET}}_{1}$	$\alpha_{E}$	${\text{Power}}_{E}$	${\mathrm{EN}}_{0}$	${\mathrm{EN}}_{1}$	$\bar{\mathrm{EN}}$	$\tilde{N}$	N
0.05	0.8	1/1.5	**Minimax**									
			Log‐rank(a)^a^	0.374	0.029	0.050	0.800	150.812	172.202	161.507	112	174
			Log‐rank(b)^b^	0.350	0.033	0.0498	0.801	152.421	175.562	163.991	105	178
			Sim_RMST^c^	0.592	0.119	0.050	0.801	129.855	166.734	148.294	98	176
			Scu_RMST^d^	0.451	0.122	0.049	0.801	103.646	121.401	112.524	74	128
			**Optimal**									
			Log‐rank(a)	0.556	0.104	0.050	0.800	135.848	177.432	156.640	95	187
			Log‐rank(b)	0.528	0.093	0.048	0.801	138.516	177.612	158.064	96	186
			Sim_RMST	0.564	0.141	0.048	0.803	127.016	169.779	148.397	83	184
			Scu_RMST	0.523	0.118	0.049	0.800	97.979	124.317	111.148	67	132
0.05	0.8	1/1.7	**Minimax**									
			Log‐rank(a)	0.325	0.028	0.050	0.800	95.625	106.020	100.823	72	107
			Log‐rank(b)	0.359	0.056	0.050	0.800	90.592	106.965	98.779	56	110
			Sim_RMST	0.508	0.106	0.049	0.801	81.639	99.327	90.483	60	104
			Scu_RMST	0.473	0.165	0.050	0.800	64.391	74.548	69.470	47	80
			**Optimal**									
			Log‐rank(a)	0.488	0.076	0.050	0.800	90.016	107.732	98.874	68	111
			Log‐rank(b)	0.467	0.088	0.050	0.802	89.370	110.961	100.165	59	116
			Sim_RMST	0.482	0.123	0.050	0.802	80.067	100.878	90.472	50	108
			Scu_RMST	0.532	0.155	0.048	0.800	62.172	77.653	69.912	43	84
0.1	0.85	1/1.5	**Minimax**									
			Log‐rank(a)	0.310	0.018	0.100	0.850	141.910	153.298	147.604	115	154
			Log‐rank(b)	0.332	0.027	0.098	0.850	136.387	150.740	143.563	105	152
			Sim_RMST	0.528	0.088	0.098	0.851	122.749	150.469	136.609	93	156
			Scu_RMST	0.493	0.134	0.097	0.850	104.838	121.683	113.261	81	128
			**Optimal**									
			Log‐rank(a)	0.358	0.042	0.100	0.850	134.088	154.312	144.200	93	157
			Log‐rank(b)	0.432	0.049	0.100	0.851	133.204	156.987	145.095	98	160
			Sim_RMST	0.419	0.078	0.098	0.852	126.141	152.057	139.099	82	158
			Scu_RMST	0.496	0.129	0.100	0.850	99.747	123.602	111.675	67	132
0.1	0.85	1/1.7	**Minimax**									
			Log‐rank(a)	0.350	0.024	0.100	0.850	89.750	94.640	92.195	80	95
			Log‐rank(b)	0.357	0.046	0.100	0.850	85.302	94.620	89.961	66	96
			Sim_RMST	0.313	0.067	0.100	0.851	82.619	89.978	86.298	62	92
			Scu_RMST	0.438	0.096	0.099	0.850	58.622	67.509	63.066	44	70
			**Optimal**									
			Log‐rank(a)	0.309	0.035	0.100	0.850	86.494	95.810	91.152	63	97
			Log‐rank(b)	0.382	0.045	0.098	0.850	84.241	96.394	90.318	62	98
			Sim_RMST	0.323	0.062	0.100	0.852	79.779	91.254	85.517	50	94
			Scu_RMST	0.422	0.104	0.100	0.854	55.128	67.840	61.484	32	72

^a^ The ${\mathrm{PET}}_{0}$, ${\mathrm{PET}}_{1}$, $\tilde{N}$ and N of Log‐rank(a) are collected from Kwak and Jung^6^ Table 1.

^b^ Log‐rank(b): Log‐rank test by grid searching critical values $\left(c_{l},c\right)$ for both stages described at Section 4.1.

^c^ Simple RMST defined in Section 4.1 comparing RMST difference between control and experimental groups only.

^d^ Sculpted RMST is the proposed method.

### 4.2 Minimax and Optimal Designs in Different Scenarios

In order to comprehensively exam the performance of our design, a series of comparative two‐stage experiments are conducted under different scenarios.

Following the notations in Section 3.4, we assume uniform accrual and censoring distribution: entry time $E_{\mathrm{ik}}∼U\left[0,a\right]$, censoring time $C_{\mathrm{ik}}∼U\left[b,a+b\right]$, where b is the minimum follow‐up time of each patient and recruit rate r=N/a. Under $H_{1}$, the survival time of patients in control group follows the exponential distribution: $T_{\mathrm{Ck}}∼\exp\left(\lambda_{0}\right)$. Under the proportional hazards assumption, survival time in experiment group $T_{\mathrm{Ek}}∼\exp\left(\lambda_{1}\right)$, where hazard ratio(HR) $\Delta =\lambda_{1}/\lambda_{0}$. Under non‐proportional hazard assumption(NPH), $T_{\mathrm{Ek}}$ follows piece‐wise exponential distribution with the survival function

$$S_{E}\left(t\right)=\exp\left\{-\Delta_{1}\cdot \lambda_{0}\left(t\wedge \mathrm{CT}\right)-\Delta_{2}\cdot \lambda_{0}{\left(t-\mathrm{CT}\right)}_{+}\right\},$$

where CT is the change time of HR. Sculpted RMST, Simple RMST and two‐stage log‐rank test are considered in the experiments. Simple RMST is the degenerated form of Sculpted RMST when $q_{1}=q_{2}=0$, which only compares the RMST difference between groups. When $\tilde{N}=N$, the performance of Simple RMST is similar to single‐stage one‐sided RMST test defined in Equation (2), where the error mainly comes from the step of grid search and Monte‐Carlo simulations.

#### 4.2.1 Comparison Under Proportional Hazards

Table 1 presents the Minimax and Optimal designs under the proportional hazards assumption, where N, $\tilde{N}$, and ${\mathrm{PET}}_{h}$ of the Log‐rank(a) are collected from Table 1 in Kwak and Jung [6]. The hazard ratio in that paper is defined as $\lambda_{C}/\lambda_{E}$ so the reciprocals are shown in our table for consistency. The total sample size of Minimax design in their paper [6] is close to the sample size of single‐stage one‐sided log‐rank test under the same α and power constraint. The patient recruiting rate is 60 per year in every setting. ${\mathrm{EN}}_{h}$ and $\bar{\mathrm{EN}}$ are calculated by the Equation (10). $\alpha_{E}$ and ${\text{Power}}_{E}$ are the empirical type I error and power of Monte‐Carlo simulation(B=5000) respectively. Under $H_{1}$, $\lambda_{E}=0.9$. The performance of Log‐rank(b), the results of grid searching the critical values of two‐stage log‐rank statistics, is close to the theoretical result of Log‐rank(a) under every case shown. The total sample size N for Simple RMST is also close to that of the log‐rank test, but it offers a slightly smaller interim sample size to reach the same power. While maintaining the stated levels of type I error and power, our Sculpted RMST has the smallest interim sample size $\tilde{N}$ and total sample size N for the Minimax design, and the smallest expected sample size under $H_{0}$ (${\mathrm{EN}}_{0}$) for the Optimal design among the three methods in all experiments. This indicates that our method is more capable of detecting treatment futile during interim analysis and has a higher inclination to stop the experiment, thereby saving resources and time. In terms of the probability of early termination (PET), ${\mathrm{PET}}_{0}$ is always positively correlated with ${\mathrm{PET}}_{1}$. The likelihood of both stopping by mistake when the treatment shows a positive effect at interim (${\mathrm{PET}}_{1}$) and stopping successfully when futile (${\mathrm{PET}}_{0}$) for Sculpted RMST is competitive to other methods. The log‐rank test is considered powerful under the proportional hazards (PH) assumption, and our Sculpted RMST method can still outperform it in terms of sample size.

#### 4.2.2 Comparison Under Early Difference Scenario

To verify the dominance of Sculpted RMST under NPH, the optimal designs for the three methods under different settings of early difference are shown in Table 2. Under $H_{1}$, the control group $\lambda_{0}=1.5$. CT = 1 year is the time at which HR changes from $\Delta_{1}$ to $\Delta_{2}=1$. τ is the cutoff time in year for RMST at final stage. The cutoff time at interim is the minimax survival time of two groups. Since we consider constant rate recruiting scenario, τ should be relatively larger when the total sample size is large, which indicates a longer study period. For all methods, the total sample size N of Optimal design would be slightly higher than Minimax design but the interim sample size $\tilde{N}$ would be lower. The ${\mathrm{PET}}_{0}$ for Optimal is larger or close to meet a lower expected sample size ${\mathrm{EN}}_{0}$. The total sample size of Simple RMST under the same α and power constraint is always smaller than that of the log‐rank test, which is consistent with the single‐stage result shown in Eaton et al. [9] under early differences. Moreover, our Sculpted RMST method can achieve the stated power with an even smaller total sample size N and interim sample size, while strictly preserving type I error and power constraints. Notably, $\tilde{N}$, N, and ${\mathrm{EN}}_{0}$ of the Sculpted RMST are nearly half of those in the log‐rank test, indicating that we could potentially save around 30% of the time and patients when the treatment is not effective. Furthermore, ${\mathrm{EN}}_{1}$ of our method is also around 2/3 of that of the log‐rank test, demonstrating that it can use only 2/3 of the resources to verify an effective treatment. In conclusion, the Sculpted RMST outperforms the other two methods in terms of sample size and expected sample size under early differences.

**TABLE 2:** Minimax and optimal two‐stage design under early difference.

α	Power	$\Delta_{1}$	τ	Method	${\mathrm{PET}}_{0}$	${\mathrm{PET}}_{1}$	$\alpha_{E}$	${\text{Power}}_{E}$	${\mathrm{EN}}_{0}$	${\mathrm{EN}}_{1}$	$\bar{\mathrm{EN}}$	$\tilde{N}$	N
0.05	0.8	0.5	2	**Minimax**									
				Log‐rank	0.519	0.040	0.050	0.801	84.286	102.480	93.383	66	104
				Sim_RMST	0.317	0.022	0.049	0.800	79.113	87.395	83.254	60	88
				Scu_RMST	0.578	0.137	0.049	0.800	54.962	66.438	60.700	44	70
				**Optimal**									
				Log‐rank	0.681	0.117	0.048	0.803	77.766	112.734	95.250	58	120
				Sim_RMST	0.522	0.100	0.049	0.800	69.554	87.700	78.627	49	92
				Scu_RMST	0.549	0.142	0.050	0.801	53.320	67.186	60.253	38	72
0.05	0.8	0.4	1.5	**Minimax**									
				Log‐rank	0.403	0.035	0.050	0.800	56.722	65.186	60.954	43	66
				Sim_RMST	0.305	0.046	0.049	0.801	45.724	49.359	47.542	36	50
				Scu_RMST	0.446	0.140	0.048	0.804	34.653	38.322	36.488	28	40
				**Optimal**									
				Log‐rank	0.469	0.069	0.049	0.801	54.879	66.079	60.479	40	68
				Sim_RMST	0.361	0.091	0.050	0.800	44.821	53.185	49.003	25	56
				Scu_RMST	0.492	0.158	0.050	0.803	34.156	40.832	37.494	24	44
0.1	0.85	0.5	1.8	**Minimax**									
				Log‐rank	0.536	0.061	0.097	0.852	76.168	93.736	84.952	59	96
				Sim_RMST	0.410	0.062	0.099	0.850	64.104	74.231	69.168	47	76
				Scu_RMST	0.471	0.131	0.097	0.850	50.109	57.257	53.683	39	60
				**Optimal**									
				Log‐rank	0.559	0.086	0.099	0.851	74.911	99.549	87.230	52	104
				Sim_RMST	0.399	0.046	0.098	0.850	62.849	76.237	69.543	40	78
				Scu_RMST	0.379	0.099	0.098	0.850	49.658	58.781	54.219	30	62
0.1	0.85	0.4	1.2	**Minimax**									
				Log‐rank	0.327	0.032	0.098	0.851	49.419	53.549	51.484	40	54
				Sim_RMST	0.316	0.046	0.097	0.851	43.897	47.399	45.648	35	48
				Scu_RMST	0.380	0.102	0.099	0.850	34.680	38.566	36.623	26	40
				**Optimal**									
				Log‐rank	0.367	0.058	0.099	0.851	49.182	56.618	52.900	34	58
				Sim_RMST	0.308	0.067	0.099	0.850	42.618	48.402	45.510	26	50
				Scu_RMST	0.340	0.121	0.100	0.850	34.112	39.197	36.655	19	42

#### 4.2.3 Comparison Under Late Difference Scenario

Late difference presents a more challenging scenario than early difference. The power of tests is typically lower than that under early difference with same setting parameters and total sample size. This is because the HR under $H_{1}$ in the early stage is close to or equal to 1, making the trial more likely to be terminated for futility during the interim period by. It results in failing to reject $H_{0}$ which leads to a significant loss of power. Therefore, the CT is set to be only 0.5 year which means that the HR between groups changes from $\Delta_{1}=1$ to $\Delta_{2}$ at half a year. $\lambda_{0}=1$, minimum follow‐up time b=1 year and patient recruit rate r=120 per year. Unlike the early difference scenario, most of the Optimal designs are the same as the Minimax designs since it is almost impossible to distinguish the difference between groups at an early stage when $\Delta_{1}=1$. Consequently, all tests require more accumulated samples and a higher number of events to make a decision at interim. Therefore, we could not always find a setting with a slightly larger N than Minimax but significantly reduced $\tilde{N}$ to achieve a smaller ${\mathrm{EN}}_{0}$. Additionally, τ would be larger as the patient recruitment period (N/r) is longer than in the early difference scenario. Similar to the early difference scenario, the Sculpted RMST provides a lower $\tilde{N}$, N, and ${\mathrm{EN}}_{0}$ in all cases shown, while preserving type I error and power. Although the gaps in sample size and expected sample size between the Sculpted RMST and the log‐rank test are smaller than those in the early difference, our method can still save around 10% of the total sample size and 20% of the expected sample size under both hypotheses compared with the log‐rank test in the late difference scenario.

#### 4.2.4 Comparison Between Single and Two‐Stage Design

To further exam the properties of two‐stage designs, we also computed the required sample size for the corresponding single‐stage designs under the same data‐generating settings as Tables 1, 2, 3. The results are summarized in Table S1. We reported the single‐stage sample size $N_{\mathrm{sg}}$ and the ratio $N_{\mathrm{sg}}/{\tilde{N}}_{\mathrm{opt}}$ relative to the interim sample size of the optimal two‐stage design. Under the late difference settings, $N_{\mathrm{sg}}/{\tilde{N}}_{\mathrm{opt}}$ is noticeably below 1 across methods. This suggests that, a two‐stage futility design may require a relatively late interim analysis to avoid premature stopping in late difference setting, whereas a single‐stage design targets only the final analysis and can achieve the same power with fewer patients.

**TABLE 3:** Minimax and optimal two‐stage design under late difference.

α	Power	$\Delta_{2}$	τ	Method	${\mathrm{PET}}_{0}$	${\mathrm{PET}}_{1}$	$\alpha_{E}$	${\text{Power}}_{E}$	${\mathrm{EN}}_{0}$	${\mathrm{EN}}_{1}$	$\bar{\mathrm{EN}}$	$\tilde{N}$	N
0.05	0.8	0.45	3	**Minimax**									
				Log‐rank	0.321	0.091	0.049	0.800	234.978	252.933	243.955	182	260
				Sim_RMST	0.324	0.126	0.048	0.801	214.460	231.307	222.883	157	242
				Scu_RMST	0.384	0.165	0.048	0.802	183.411	197.440	190.426	144	208
				**Optimal**									
				Log‐rank	0.373	0.117	0.049	0.800	231.956	253.972	242.964	178	264
				Sim_RMST	0.355	0.132	0.050	0.801	214.032	234.084	224.058	156	246
				Scu_RMST	0.384	0.165	0.048	0.802	183.411	197.440	190.426	144	208
0.05	0.8	0.4	2.5	**Minimax**									
				Log‐rank	0.344	0.131	0.049	0.800	205.576	220.727	213.152	159	230
				Sim_RMST	0.306	0.111	0.048	0.800	197.997	211.994	204.995	148	220
				Scu_RMST	0.334	0.170	0.049	0.800	173.638	183.606	178.622	133	194
				**Optimal**									
				Log‐rank	0.344	0.131	0.049	0.800	205.576	220.727	213.152	159	230
				Sim_RMST	0.306	0.111	0.048	0.800	197.997	211.994	204.995	148	220
				Scu_RMST	0.372	0.178	0.048	0.802	173.422	186.226	179.824	132	198
0.1	0.85	0.45	3	**Minimax**									
				Log‐rank	0.300	0.097	0.096	0.851	226.400	241.045	233.722	176	248
				Sim_RMST	0.336	0.114	0.099	0.850	214.464	231.306	222.885	164	240
				Scu_RMST	0.343	0.135	0.097	0.852	195.362	209.090	202.226	152	218
				**Optimal**									
				Log‐rank	0.335	0.122	0.096	0.851	226.221	245.142	235.681	167	256
				Sim_RMST	0.305	0.112	0.098	0.853	214.837	232.050	223.444	153	242
				Scu_RMST	0.348	0.136	0.100	0.852	196.350	210.752	203.551	152	220
0.1	0.85	0.4	2.5	**Minimax**									
				Log‐rank	0.300	0.112	0.098	0.850	206.400	219.965	213.182	156	228
				Sim_RMST	0.308	0.112	0.095	0.854	201.178	215.742	208.460	150	224
				Scu_RMST	0.332	0.141	0.096	0.852	183.428	195.283	189.356	142	204
				**Optimal**									
				Log‐rank	0.300	0.112	0.098	0.850	206.400	219.965	213.182	156	228
				Sim_RMST	0.308	0.112	0.095	0.854	201.178	215.742	208.460	150	224
				Scu_RMST	0.332	0.141	0.096	0.852	183.428	195.283	189.356	142	204

### 4.3 Effect of Interim Sample Size on Power

To better understand the nature of our design, we further clarify the relationship between the power of the two‐stage design and interim sample size when total sample size N is fixed.

**FIGURE 2:**
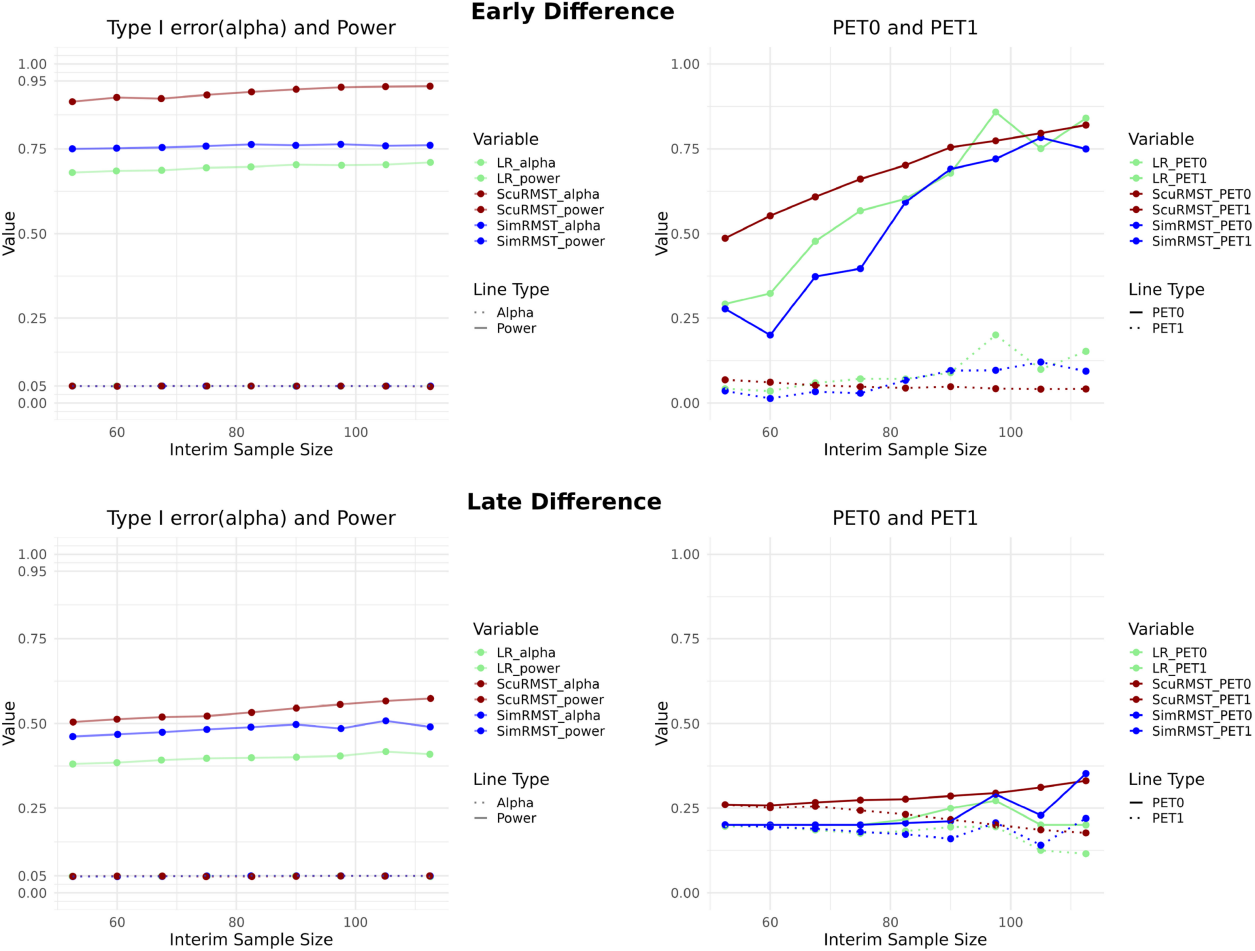
Performance of log‐rank, Simple RMST and Sculpted RMST under non‐proportional hazards assumption. Settings: N=150,accrual rater=100,b=1,CT=0.6. Hazard of control group $\lambda_{0}=1.5$. Cut‐off time τ=2.5. Hazard ratio of early difference: $\Delta_{1}=0.5,\;\Delta_{2}=1$. Late difference: $\Delta_{1}=1,\;\Delta_{2}=0.5$. Legends show Log‐rank test, Simple RMST and Sculpted RMST. PET is the probability of early termination.

Take early difference and late difference as examples. When recruiting patients at a constant rate (*r* = 60/year). Figure 2 depicts the power curve versus different interim sample size $\tilde{N}$ meaning that conducting interim analysis at calender time $\tilde{N}/r$ in two NPH settings (Monte‐Carlo B=10000 for each $\tilde{N}$). The contours of lines in both scenarios are similar. The empirical α are strictly controlled below 0.05 in all tests. The power of the three methods are not significantly affected by the interim sample size. The power of Sculpted RMST significantly outweighs the other two methods and slightly increases with interim sample size. In the early difference scenario, the ${\mathrm{PET}}_{0}$ of Sculpted RMST is way larger than the other two methods at early interim phase while the ${\mathrm{PET}}_{1}$ is also competitive. This indicates that Sculpted RMST can detect futility and stop the trial more efficiently with smaller interim sample size. In the late difference scenario, ${\mathrm{PET}}_{0}$ and ${\mathrm{PET}}_{1}$ are similar across all methods since the hazard ratio between the two groups is 1 during the early stage. Since the hazard ratio turns to 0.5 after 0.6 years and additional evidences are collected to reject $H_{0}$ as the trial goes on, ${\mathrm{PET}}_{1}$ of all methods decreases as interim sample size raises in late difference. Our method demonstrates a higher probability of early termination compared to the other two methods when the null hypothesis holds (${\mathrm{PET}}_{0}$) in the late difference scenario. This means that our method have a greater likelihood of stopping the experiment early, thereby saving resources and time under $H_{0}$. Although the sculpted RMST slightly sacrifices the PET under $H_{1}$, the gap between $\mathrm{LR}\_{\mathrm{PET}}_{0}$ and $\text{Sculpted}\_{\mathrm{PET}}_{0}$ surpasses their ${\mathrm{PET}}_{1}$ difference and the ${\mathrm{PET}}_{1}$ gap narrows as the $\tilde{N}$ rises. Thus in summary, the Sculpted RMST performs better than the other two under these NPH settings in terms of power and ${\mathrm{PET}}_{0}$.

### 4.4 Visualization of Two‐Stage RMST Critical Regions

**FIGURE 3:**
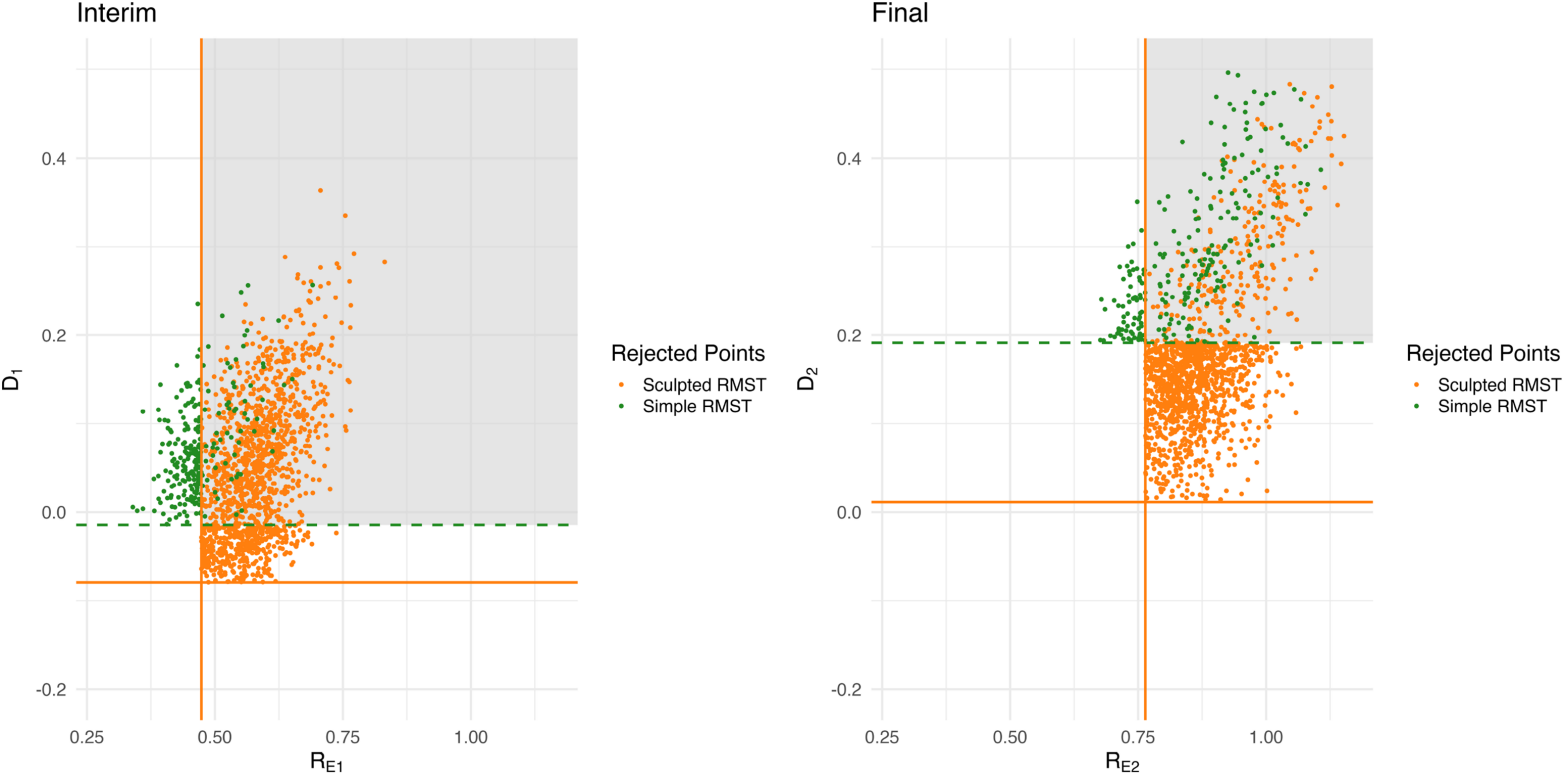
The data points in overall rejection region of $H_{1}$ in 10 000 simulated trials. Each dot in subplot A representing a pair of $\left({\hat{R}}_{E1},{\hat{R}}_{C1}\right)$, which is one simulated trial in interim stage, has a corresponding dot with the same color in subplot B indicating the final stage of the same trial. The shaded regions are the critical regions interaction of simple and Sculpted RMST. Lines are decision boundaries maximizing the empirical power of two methods in two stages.

A phase II trial requires a smaller sample size because it primarily aims to assess efficacy and safety rather than provide definitive evidence for regulatory approval. In this study, it was observed that Sculpted RMST achieves a similar power level with a smaller total sample size compared to Simple RMST. This indicates that the empirical power of Sculpted RMST consistently exceeds that of Simple RMST under the same sample size and type I error constraints. To clarify the differences in power, it would be beneficial to visualize the rejection regions of Simple and Sculpted RMST. Figure 3 shows the rejection points out of 10 000 simulated trials under $H_{1}$. We use the first setting of PH in Table 1, where the hazard of two groups $\lambda_{E}=0.9,\;\lambda_{C}=0.9\times 1.7$ with exponential survival function. The other 10 000 trials are simulated under $H_{0}:\lambda_{E}=\lambda_{C}=0.9\times 1.7$. Overall sample size N=100, interim $\tilde{N}=60$, accrual rate r=60 patients per year. Cut off time τ=2.5 years. Then the critical values maximizing empirical power are obtained from the searching procedure in Section 3.4 under α=0.05. The empirical type I error of the Simple and Sculpted RMST are 0.0498 and 0.0497 respectively, while the empirical power are 0.8221 and 0.9093 respectively. To highlight the difference in empirical power, the simulated trials that are announced effective under $H_{1}$ by both methods, the overlapping dots, are omitted from Figure 3. In plot A, there are 197 green dots and 1069 orange dots and same as subplot B. The difference in empirical power is indicated by (1069−197)/10000=0.9093−0.8221=0.0872. Firstly, we observe that many green and orange dots are present in the shaded region during the interim phase, while most orange dots move out of the shaded region at final stage. In the interim phase, there are 126 green dots and 38 orange dots outside the shaded region. However, in the final phase, the number of dots outside the shaded region changes to 76(green) and 1046(orange) respectively. This suggests that 1008 orange dots move out of the shaded region from interim to final, which is the main source of additional power of Sculpted RMST compared with Simple RMST. More precisely, there are more simulated trials with small ${\hat{D}}_{2}$ but large ${\hat{R}}_{E2}$ at final stage fail to reject $H_{0}$ due to the stringent boundary of ${\hat{D}}_{2}$ in Simple RMST (horizontal dotted line). All dots are generated under $H_{1}$. The small ${\hat{D}}_{2}$ of 1046 orange dots is attributed to the relatively large ${\hat{R}}_{C2}$ in the control group. Our motivation of sculpting the critical region can be also interpreted as ensuring that trials with a sufficiently long life expectancy in the experimental group are correctly identified as effective. Therefore, the movement of the dots supports this idea.

In addition, we also visualize the rejection region using the $\left({\hat{R}}_{E},{\hat{R}}_{C}\right)$ plane. Figures S1–S3 in the the Supporting Information: Appendix show scatter plots and two‐dimensional density projections of $\left({\hat{R}}_{E},{\hat{R}}_{C}\right)$ under the same setting as Figure 3. These representations are more clinically intuitive because both axes are arm‐specific RMSTs.

### 4.5 Robustness

**FIGURE 4:**
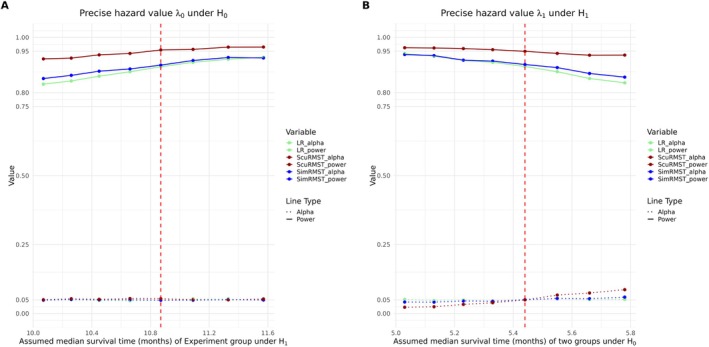
Robustness under different λ. Subplot A is simulating a precise assumption of $\lambda_{0}$ but the real $\lambda_{1}$ deviates from assumed value. Similarly, subplot B is the performance when the assumed $\lambda_{1}$ is accurate but real $\lambda_{0}$ drifts. For ease of understanding, the hazard ratio is transformed to median survival time in months by log (2)/hazard ratio * 12.

The sculpted critical region is more stringent and sensitive to the assumed parameters in the hypothesis. The global robustness about the type I error control is discussed in Litwin et al. [3] with binary endpoint. In time‐to‐event endpoint with proportional hazards, it is appropriate to assume that

$$\begin{array}{ll} & H_{0}:\;\lambda_{E}=\lambda_{C}=\lambda_{0},\\ & H_{1}:\;\lambda_{C}=\lambda_{0},\;\lambda_{E}=\lambda_{1}, \end{array}$$

where $\lambda_{E}$ and $\lambda_{C}$ are the hazards of the experimental and control groups, respectively. $\lambda_{0}$ and $\lambda_{1}$ are the corresponding assumed values in the design phase. The robustness of three tests are shown in Figure 4. We use exactly the same setting as that in Section 4.4, namely, $\lambda_{0}=1.5$, $\lambda_{1}=1.5^{*}0.5=0.75,\;N=90,\;\tilde{N}=45,\;r=60,\;\tau =2$. The empirical type I error $\left(\alpha_{E}\right)$, empirical power $\left({\text{power}}_{E}\right)$, ${\mathrm{PET}}_{0}$ and ${\mathrm{PET}}_{1}$ are estimated after searching for the critical values maximizing empirical power, which are displayed with dotted orange lines of two subplots. The critical values under this setting are saved and then plugged into the survival data simulated under different $\lambda_{1}$ (subplot A) or $\lambda_{0}$ (subplot B). 10 000 pairs of experimental and control groups survival data are generated under each $\lambda_{0}$ or $\lambda_{1}$ while preserving the hazard of the other group.

In our hypothesis setting, the hazard of control group under $H_{1}$ is equal to the hazard of two groups under $H_{0}$. So the change of $\lambda_{0}$ will affect $H_{0}$ and the HR in $H_{1}$ simultaneously. In subplot A, the change of $\lambda_{1}$ will only affect the HR in $H_{1}$ but not $H_{0}$. So the $\alpha_{E}$ curve of three methods are nearly horizontal. Besides, the drift of Sculpted RMST ${\text{power}}_{E}$ is similar to that of the other two methods. In subplot B, $\alpha_{E}$ of log‐rank test remains almost constant across different $\lambda_{0}$ and Simple RMST has a slight downtrend as $\lambda_{0}$ increases. The variation of Sculpted RMST $\alpha_{E}$ is slightly larger than other two methods. This indicates that the actual type I error of the Sculpted RMST will deviate slightly more from the expected stated level compared to the other two methods if the assumed hazard in control group is inaccurate. This is a trade‐off of critical region sculpting. The additional term of $R_{E}$ in decision rule allows for more precise region cutting to satisfy α and power constraint as shown in Figures S2 and S3. However, the number of points in the rejection region becomes more sensitive to the drift of the distribution parameter of RMST, leading to an increase of type I error when $\lambda_{0}$ is overestimated during design phase.

One practical approach to deal with the imprecise guess of $\lambda_{0}$ is conducting a series of experiments with hazard in the vicinity of the assumed one. For instance, if we assume $\lambda_{1}=0.75$, we can search for the optimal design when $\lambda_{0}\in \left\{1.4,1.45,1.5,1.55,1.60\right\}$ under α=0.05. In this way, the change of sample size and empirical type I error can still be managed that the researchers can determine how conservative the trial design would be. Similar approach is used to address robustness issue in Litwin et al. [3].

As in Jung [2], enhancing robustness over a composite null typically increases the required sample size. We emphasize that the grid over $\lambda_{0}$ is used for sensitivity analysis rather than as part of the primary design optimization. If uniform type I error control is desired over a plausible range of $m_{0}$ values, a conservative choice is to take the largest total sample size. Besides, Table S2 further explores this issue by re‐optimizing the minimax design under a shifted baseline hazard $\lambda_{0}^{⋆}=0.9\lambda_{0}$. The resulting N may increase or decrease across scenarios because changing $\lambda_{0}$ affects both the RMST effect size over [0,τ] and the variance of RMST estimators under censoring.

### 4.6 Trial Example

In this section, three methods are applied to a real phase II trial data to determine the Minimax design that minimizes N following the procedure used in previous experiments. The considered example is a multicentre phase II 1:1 randomized controlled trial that determine if Atezolizumab (experimental group) can improve survival compared with docetaxel (control group) in patients with previously treated NSCLC [17]. The primary endpoint is overall survival (OS). In the study, a total of 287 patients were recruited over 8 months, where 144 patients were randomly allocated to receive atezolizumab, while 143 patients were assigned to receive docetaxel. The final analysis is conducted when 173 deaths occurred using log‐rank test with a two‐sided α=0.0488, power=0.823. The estimator for the median OS in the experimental and control groups are 12.6 and 9.7 months, respectively by Kaplan–Meier approach. The estimated HR = 0.73 by stratified Cox regression models.

We first applied the proportional hazards assumption with exponential survival under $H_{0}:\lambda_{E}=\lambda_{C}=\log\left(2\right)/9.7$ against $H_{1}:\lambda_{C}=\log\left(2\right)/9.7,\;\lambda_{E}=\log\left(2\right)/12.6$. log(2) comes the property within exponential survival: if S(t)=exp(−λt), median survival is log(2)/λ. Simulated data are generated according to this assumption, 8‐month accrual and minimum 13‐month follow‐up time stated in trial [17]. Then the Minimax design of three methods are found under (α=0.05,power=0.8) and shown in the first three columns in Table 4. The cut‐off time τ=20 months. The Sculpted RMST can provide significantly lower ${\mathrm{EN}}_{0}$, $\tilde{N}$, and N compared to both the log‐rank test and Simple RMST under PH, while maintaining the type I error and power levels. Furthermore, our method has significantly larger ${\mathrm{PET}}_{0}$, indicating a higher probability of saving time and resources when treatment is found to be ineffective at interim.

**TABLE 4:** Minimax design in atezolizumab versus docetaxel trial [17] under PH.

Assumption	Method	$\alpha_{E}$	${\text{Power}}_{E}$	${\mathrm{PET}}_{0}$	${\mathrm{PET}}_{1}$	${\mathrm{EN}}_{0}$	${\mathrm{EN}}_{1}$	$\bar{\mathrm{EN}}$	$\tilde{N}$	N
PH	Log‐rank	0.049	0.806	0.310	0.065	297.916	326.387	312.152	218	334
	Sim_RMST	0.050	0.802	0.328	0.063	292.948	321.229	307.089	222	328
	Scu_RMST	0.049	0.800	0.433	0.134	183.594	208.710	196.152	136	220

## 5 Discussion

In this study, RMST with a sculpted critical region is applied to two‐stage double‐arm trial designs. Distinguished from the traditional RMST difference test, the sculpted critical region requires not only a large RMST difference between groups $\hat{D}\left(\tau \right)$ but also a significantly large value in experimental group ${\hat{R}}_{E}\left(\tau \right)$ to continue the trial. The cut‐off time τ for RMST estimation at the final stage should be prespecified during the design phase. This ensures that the RMST result can be interpreted as the life expectancy up to time τ. The estimation of critical values is not restricted by the distribution of survival data. Besides, the decision boundaries between two stages is controlled by the probability control function f about interim sample size, which can prevent stopping prematurely with too small interim sample size and determine suitable critical values based on simulated survival data.

Under PH, the log‐rank test is considered to be a powerful test, but our Sculpted RMST can still outperform it in terms of smaller total sample size, early interim study and smaller expected sample size under the same α and power constraint. Under NPH, our method outperforms log‐rank test and RMST without sculpting in different settings. The performance gap between Sculpted RMST and other methods under early difference is the largest, which can possibly save half of time and patients to achieve the same power level. Experiments reveal the sculpted region indeed optimizes the original RMST test based on difference only. The motivation behind sculpting is to reduce the type I error rate α from incorrectly rejecting $H_{0}$ when the estimated RMST in the control group is low. In clinical practice, it is essential to control the type I error rate at a certain level. As shown in Figure 3, the sculpted region ensures that trials with a sufficiently long life expectancy in the experimental group are correctly identified as effective, thereby effectively increasing the power. In addition to these advantages, our method can be easily adapted to sequential trial design with a series of $f\left(N_{i}\right)$ constraints in the critical values solving steps. However, the trial would become more stringent as the number of interim analyses increases. Overly strict phase II trials are not always suggested since the initial evidence of efficacy in early phase would be valuable for the following trials. Thus, we only consider two‐stage trial design.

Despite a trade‐off in global robustness of α across unexpected hazards of control group, researchers can overcome this by conducting a series of simulated trials with more conservative assumption on hazard in the vicinity of the stated one. The range of empirical type I error rate can still be controlled. Litwin et al. [3] mentioned that the robustness effect can be traced to the decision boundary of $R_{E2}>q_{2}$ at final stage as $q_{2}$ will become less relevant to the accumulated measurement of experiment group at final stage. So another feasible method is to apply an additional parameter λ and change our threshold function to $f\left(\tilde{N}\right)=\exp\left(-\gamma \cdot {\left(\frac{\tilde{N}}{N}\right)}^{\lambda }\right)$. This approach provides more relaxed restrictions, leading to more possible combinations of critical values that satisfy the α and power constraints. The combination with the largest $q_{2}$ value among all settings that achieve empirical power higher than the stated $1-\beta_{0}$ level can be selected. However, listing all possible Type I error rates as the assumed parameter drifts is more straightforward and user‐friendly for clinicians to better balance robustness and power. Moreover, one may concern that if both RMST values in the experimental and control groups are overestimated under the alternative hypothesis, $\hat{D}\left(\tau \right)$ is large but ${\hat{R}}_{E}\left(\tau \right)$ is not large enough to reject $H_{0}$. In fact, treatment effect that is lower than expected should have been considered failure in the actual clinical trials. However, this scenario is also difficult to be detected relying solely on the RMST difference whereas the sculpted region can achieve. Under a certain $\hat{D}\left(\tau \right)$ level, the trial with low ${\hat{R}}_{E}\left(\tau \right)$ should not be included in the rejection region of $H_{0}$. Thus, it also proves that our motive of driving only trials with low ${\hat{R}}_{C}\left(\tau \right)$ away from the rejection region is appropriate. This case is also discussed in Litwin et al. [3], where it is mentioned that the sculpted region of success rate with binary endpoints was being used widely at Fox Chase Cancer Center in Philadelphia. Thus, it is worth believing that the sculpted approach is reasonably reliable in real trials.

## Funding

The work described in this paper was substantially supported by a grant from Hong Kong Metropolitan University (Project Reference No. HE‐FRSF/2025/06).

## Conflicts of Interest

The authors declare no conflicts of interest.

## Supporting information


**Data S1:** Supporting Information.

## Data Availability

The data that support the findings of this study are openly available in Sculpted RMST Two Stages Double‐arm Clinical Trial Design at https://github.com/garcia‐ho/RMST_Code.git.
