# Supplementary material for: Two‐Stage Double‐Arm Trial Optimal Design of Restricted Mean Survival Time With Sculpted Critical Region
Source: Stat Med. 2026 May 17;45:e70589. doi: 10.1002/sim.70589 (PMC13180504; doi:10.1002/sim.70589)
Supplement: Supplementary file 1 — Data S1: Supporting Information. [file SIM-45-0-s001.pdf]

Supplementary Appendix

1 Mean and Variance of $\hat{R}_{Ei} \mid \hat{D}_i$

$(\hat{R}_{Ei}, \hat{D}_i)$ follows a bivariate normal distribution asymptotically. We assume the marginal distribution is $\hat{R}_{Ei} \sim N(\mu_{E_i}, \sigma^2_{E_i})$ and $\hat{D}_i \sim N(\mu_{D_i}, \sigma^2_{D_i})$ The correlation coefficient is denoted as ρ_i. The conditional distribution of $\hat{R}_{Ei} \mid \hat{D}_i = m_i$ is also normal asymptotically, which is:

$$\hat{R}_{Ei} \mid \hat{D}_i = m_i \sim N\left(\mu_{E_i} + \rho_i \frac{\sigma_{E_i}}{\sigma_{D_i}}(m_i - \mu_{D_i}), (1-\rho_i^2)\sigma^2_{E_i}\right). \tag{1}$$

Besides, $\hat{D}_i \mid \hat{D}_i > m_i$ follows a truncated normal distribution and its mean and variance can be derived as

$$\begin{aligned} \mathbb{E}[\hat{D}_i \mid \hat{D}_i > m_i] &= \mu_{D_i} + \sigma_{D_i} \frac{\phi(\theta)}{1-\Phi(\theta)}, \\ \mathrm{Var}(\hat{D}_i \mid \hat{D}_i > m_i) &= \sigma^2_{D_i}\left[1 - \frac{\theta \cdot \phi(\theta)}{1-\Phi(\theta)} - \left(\frac{\phi(\theta)}{1-\Phi(\theta)}\right)^2\right] \end{aligned} \tag{2}$$

where:

$$\theta = \frac{m_i - \mu_{D_i}}{\sigma_{D_i}}$$

$\phi(\theta)$ is the standard normal probability density function evaluated at θ, and $\Phi(\theta)$ is the standard normal cumulative distribution function evaluated at θ. Combining the mean value at eq. (1), we have

$$\begin{aligned} \mathbb{E}[\hat{R}_{Ei} \mid \hat{D}_i > m_i] &= \mathbb{E}[\mathbb{E}[\hat{R}_{Ei} \mid \hat{D}_i] \mid \hat{D}_i > m_i] \\ &= \mathbb{E}\left[\mu_{E_i} + \rho_i \frac{\sigma_{E_i}}{\sigma_{D_i}}(\hat{D}_i - \mu_{D_i}) \mid \hat{D}_i > m_i\right] \\ &= \mu_{E_i} + \rho_i \frac{\sigma_{E_i}}{\sigma_{D_i}}\left(\mathbb{E}[\hat{D}_i \mid \hat{D}_i > m_i] - \mu_{D_i}\right) \\ &= \mu_{E_i} + \rho_i \sigma_{E_i} \frac{\phi(\theta)}{1-\Phi(\theta)}. \end{aligned} \tag{3}$$

Moreover, the conditional variance is given by:

$$\mathrm{Var}(\hat{R}_{Ei} \mid \hat{D}_i > m_i) = \mathbb{E}[\mathrm{Var}(\hat{R}_{Ei} \mid \hat{D}_i) \mid \hat{D}_i > m_i] + \mathrm{Var}[\mathbb{E}(\hat{R}_{Ei} \mid \hat{D}_i) \mid \hat{D}_i > m_i].$$

From the variance at eq. (1), $\mathrm{Var}(\hat{R}_{Ei} \mid \hat{D}_i = m_i) = (1-\rho_i^2)\sigma^2_{E_i}$. Since this variance is constant with respect to D, the expectation of this variance given $D > m$ is simply:

$$\mathbb{E}[\mathrm{Var}(\hat{R}_{Ei} \mid \hat{D}_i = m_i)] = (1-\rho_i^2)\sigma^2_{E_i}.$$

The second term of the conditional variance can be simplified as:

$$\begin{aligned}\mathrm{Var}(\mathbb{E}[\hat{R}_{Ei} \mid \hat{D}_i] \mid \hat{D}_i > m_i) &= \mathrm{Var}\left(\mu_{E_i} + \rho_i \frac{\sigma_{E_i}}{\sigma_{D_i}}(\hat{D}_i - \mu_{D_i}) \mid \hat{D}_i > m_i\right) \\ &= \left(\rho_i \frac{\sigma_{E_i}}{\sigma_{D_i}}\right)^2 \mathrm{Var}(\hat{D}_i \mid \hat{D}_i > m_i)\end{aligned}$$

Combining the variance at eq. (2), We have:

$$\mathrm{Var}(\hat{R}_{Ei} \mid \hat{D}_i > m_i) = (1-\rho_i^2)\sigma_{E_i}^2 + \left(\rho_i \frac{\sigma_{E_i}}{\sigma_{D_i}}\right)^2 \cdot \sigma_{D_i}^2 \left[1 - \frac{\theta \cdot \phi(\theta)}{1-\Phi(\theta)} - \left(\frac{\phi(\theta)}{1-\Phi(\theta)}\right)^2\right] \tag{4}$$

2 Visualization of Rejection Regions

Figure S1 is plotted under the same setting of Figure 3 in the main script. In the $(\hat{R}_E, \hat{R}_C)$ plane, the Simple RMST rule $\hat{D}_i = \hat{R}_{Ei} - \hat{R}_{Ci} > m_i$ corresponds to the region below the slanted boundary $\hat{R}_{Ci} = \hat{R}_{Ei} - m_i$, whereas the Sculpted RMST additionally requires $\hat{R}_{Ei} > q_i$, shown as the vertical boundary. Consistent with the $(\hat{R}_E, \hat{D})$ display in the main text, we observe that many green and orange dots satisfy both boundaries at the interim analysis, while a substantial proportion of orange dots no longer satisfy the Simple RMST boundary at the final stage. Specifically, at the final analysis there are many simulated trials with sufficiently large $\hat{R}_{E2}$ that fall above the Simple RMST slanted boundary because $\hat{R}_{C2}$ is also large but the between-arm difference $\hat{D}_2$ is not large enough. These trials are therefore classified as ineffective by Simple RMST despite exhibiting a relatively long life expectancy in the experimental arm. In contrast, the sculpted rule retains such trials when $\hat{R}_{E2}$ exceeds the prespecified threshold. This aligns with our motivation that treatment effectiveness should not be driven solely by an unusually small control RMST. This geometric view illustrates that the additional power gain of the Sculpted RMST is largely attributable to trials with large $\hat{R}_{E2}$ but also large $\hat{R}_{C2}$, which are excluded by the Simple RMST rule due to its single slanted boundary on $\hat{D}_2$.

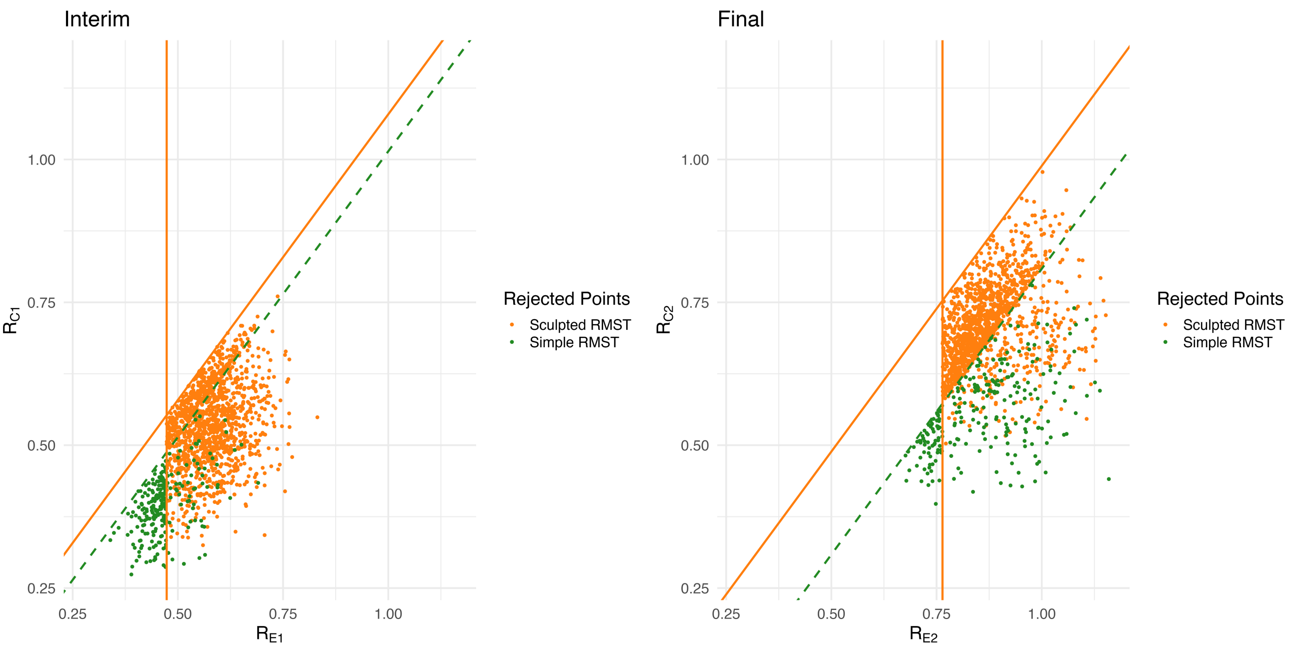

Figure S1: The joint density of RMST values $(\hat{R}_{E1}, \hat{R}_{C1})$, $(\hat{R}_{E2}, \hat{R}_{C2})$ under H_0 and H_1 are projected onto a 2-dimensional plane. The green dotted lines represent the decision boundaries of Simple RMST, while the orange solid lines are those of Sculpted RMST.

Joint Density of RMSTs

The two projections in fig. S2 and fig. S3 provide complementary geometric interpretations of the same rejection rule. In the $(\hat{R}_E, \hat{D})$ plane, the sculpted rule is shown directly as the intersection $\hat{D}_i > m_i$ and $\hat{R}_{Ei} > q_i$, so the shaded regions represent the critical regions determined by a horizontal boundary on $\hat{D}_i$ and a vertical boundary on $\hat{R}_{Ei}$. In the $(\hat{R}_E, \hat{R}_C)$ plane, the Simple RMST rule $\hat{D}_i = \hat{R}_{Ei} - \hat{R}_{Ci} > m_i$ corresponds to a slanted boundary $\hat{R}_{Ci} = \hat{R}_{Ei} - m_i$. The sculpted rule adds the same vertical boundary $\hat{R}_{Ei} = q_i$. In both plots, the volumes of the green patterns within the shaded regions represent $P(\hat{D}_i > m_i \cap \hat{R}_{Ei} > q_i \mid H_1)$, while the volumes of the orange patterns within the shaded regions represent $P(\hat{D}_i > m_i \cap \hat{R}_{Ei} > q_i \mid H_0)$. These values are positively correlated to power and type I error, respectively. Compared with the Simple RMST rule based only on $\hat{D}_i > m_i$, the additional boundary on $\hat{R}_{Ei}$ provides extra flexibility to shape the rejection region: it can retain trials with sufficiently large $\hat{R}_{Ei}$ while trimming regions that are more likely under H_0, thereby increasing the probability mass captured under H_1 while controlling the mass under H_0. This

illustrates the mechanism by which the sculpted critical region improves power under the same type I error constraint, and the $(\hat{R}_E, \hat{R}_C)$ projection offers a clinically intuitive view of the same phenomenon in terms of the two arm-specific RMSTs.

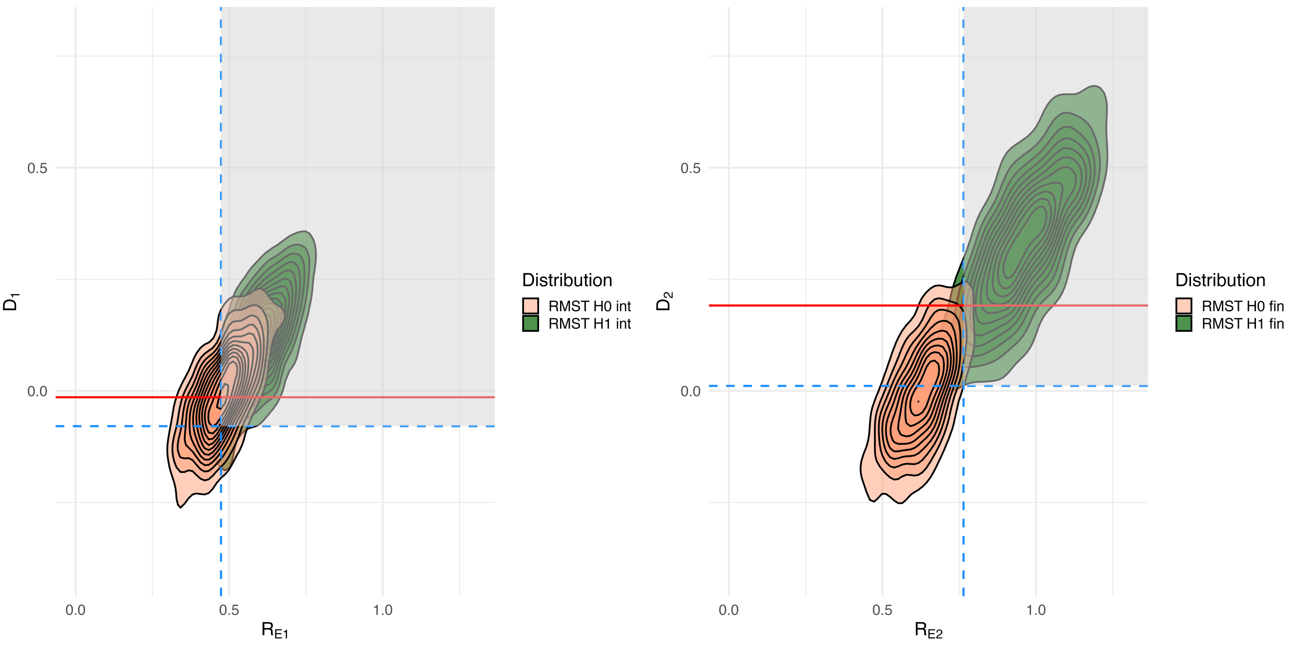

Figure S2: The joint density of RMST values $(\hat{R}_{E1}, \hat{D}_1)$ and $(\hat{R}_{E2}, \hat{D}_2)$ under H_0 and H_1 are projected onto a 2-dimensional plane. The blue dotted lines represent the decision boundaries of Sculpted RMST, while the red solid lines are those of Simple RMST. The shaded regions indicate the critical regions for Sculpted RMST.

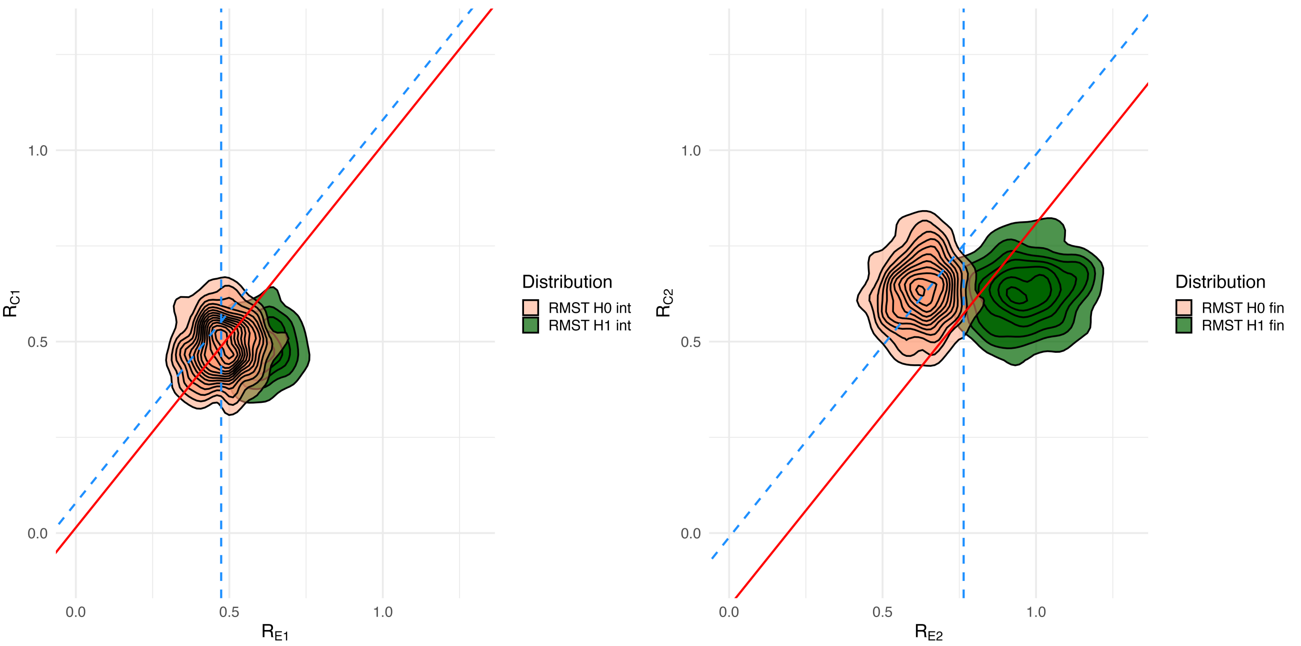

Figure S3: The joint density of RMST values $(\hat{R}_{E1}, \hat{R}_{C1})$ and $(\hat{R}_{E2}, \hat{R}_{C2})$ under the same setting as fig. S2

3 Comparison between Single and Two-stage Design

The single-stage required sample sizes reported in table S1 are obtained under the same data generating mechanisms as those used for the two-stage designs in Table 1, Table 2, and Table 3 in the main script. Δ refers to Δ_1 in early difference setting and Δ_2 in late difference. The sample size of single-stage Log-rank test is obtained by Monte-Carlo simulation under the data generating mechanism described in Section 4.1, which is analogous to Log-rank(b) in Table 1 of the main script. The single-stage sample size N_{sg} for each method is determined as the smallest N that satisfies the (α, power) constraints using the Monte-Carlo simulation. We compare the single-stage required sample size N_{sg} with the interim sample size $\tilde{N}_{\text{opt}}$ of the corresponding two-stage optimal design because $\tilde{N}$ represents the amount of information accrued at the interim stage. This ratio quantifies how early the two-stage design attempts to make a futility decision relative to a definitive single-stage conclusion under the same accrual, censoring, and follow-up settings.

Under the proportional hazards (PH), the ratio $N_{\text{sg}}/\tilde{N}_{\text{opt}}$ is generally close to 1. This indicates that the minimum single-stage sample size required to achieve the target power is similar to the sample size at which the two-stage design schedules its interim assessment. In contrast, in the early-difference settings, table S1 shows that the required single-stage sample size N_{sg} is consistently smaller than the interim sample size $\tilde{N}_{\text{opt}}$ This pattern reflects a key feature of two-stage futility designs that the interim sample size is not chosen to mimic a one-stage test. It provides a sufficiently reliable interim screening rule while preserving overall power. In order to keep PET_1 low and avoid prematurely stopping an effective treatment, the optimal two-stage design may schedule the interim analysis at a relatively late information time, even when the treatment effect emerges early. However, the single-stage design only targets the final analysis and can achieve the desired power with fewer total patients.

For the late-difference settings, $N_{\text{sg}}/\tilde{N}_{\text{opt}}$ becomes even smaller. The main reason is that early futility termination is intrinsically difficult when the treatment effect is delayed. The interim statistics under H_1 resemble those under H_0 in the early follow-up period. So a two-stage design that aims to preserve power must schedule the interim analysis relatively late, resulting in a large $\tilde{N}$. By contrast, the single-stage design is evaluated at the final analysis time and can accrue the late-emerging treatment benefit without facing an interim requirement. This phenomenon aligns with existing observations in Huang and Kuan[1]. Under non-PH scenarios with late separation, RMST-based inference can be competitive or superior when the truncation time is chosen to capture the late separation.

Table S1: Single-stage required sample sizes under different settings

α	**Power**	Δ	τ	**Method**	α_E	Power$_E$	$N_{\mathbf{sg}}$	$\tilde{N}_{\mathbf{opt}}$	$N_{\mathbf{sg}}/\tilde{N}_{\mathbf{opt}}$
**Proportional Hazards**									
				Log-rank	0.0496	0.8126	104	96	1.083
0.05	0.8	1/1.5	2.5	Sim-RMST	0.0496	0.8096	102	83	1.229
				Scu-RMST	0.0493	0.8184	66	67	0.985
				Log-rank	0.0495	0.8110	68	59	1.153
0.05	0.8	1/1.7	2.5	Sim-RMST	0.0492	0.8002	64	50	1.280
				Scu-RMST	0.0494	0.8036	42	43	0.977
				Log-rank	0.0967	0.8532	92	98	0.939
0.1	0.85	1/1.5	2.5	Sim-RMST	0.0973	0.8548	92	82	1.122
				Scu-RMST	0.0952	0.8738	60	67	0.896
				Log-rank	0.0963	0.8634	60	62	0.968
0.1	0.85	1/1.7	2.5	Sim-RMST	0.0970	0.8550	58	50	1.160
				Scu-RMST	0.0996	0.8608	36	32	1.125
**Early Difference**									
				Log-rank	0.0488	0.8192	56	58	0.966
0.05	0.8	0.5	2	Sim-RMST	0.0491	0.8016	48	49	0.980
				Scu-RMST	0.0498	0.9158	36	38	0.947
				Log-rank	0.0494	0.8021	30	40	0.750
0.05	0.8	0.4	1.5	Sim-RMST	0.0492	0.8128	26	25	1.040
				Scu-RMST	0.0498	0.8168	18	24	0.750
				Log-rank	0.0985	0.8636	46	52	0.885
0.1	0.85	0.5	1.8	Sim-RMST	0.0983	0.8512	38	40	0.950
				Scu-RMST	0.0994	0.8794	24	30	0.800
				Log-rank	0.0982	0.8632	26	34	0.765
0.1	0.85	0.4	1.2	Sim-RMST	0.0998	0.8506	22	26	0.846
				Scu-RMST	0.0994	0.8776	14	19	0.737
**Late Difference**									
				Log-rank	0.0493	0.8001	120	178	0.674
0.05	0.8	0.45	3	Sim-RMST	0.0498	0.8174	128	156	0.821
				Scu-RMST	0.0492	0.8323	90	144	0.625
				Log-rank	0.0490	0.8246	110	159	0.692
0.05	0.8	0.4	2.5	Sim-RMST	0.0496	0.8058	118	148	0.797
				Scu-RMST	0.0492	0.8032	80	132	0.606
				Log-rank	0.0979	0.8656	114	167	0.683
0.1	0.85	0.45	3	Sim-RMST	0.0980	0.8542	114	153	0.745
				Scu-RMST	0.0998	0.8876	86	152	0.566
				Log-rank	0.0992	0.8624	98	156	0.628
0.1	0.85	0.4	2.5	Sim-RMST	0.0988	0.8554	102	150	0.680
				Scu-RMST	0.0982	0.8538	72	142	0.507

4 Robustness Discussion based on Minimax Design

We conducted an additional simulation study to examine how the Minimax two-stage design changes when the the null hypothesis is misspecified. The same data-generating mechanisms as the first two settings in each of Tables 1–3 are applied. Specifically, we replaced the design-point control hazard by $\lambda_0^\star = 0.9\,\lambda_0$ while keeping the accrual and censoring settings, follow-up time, and cut-off time τ. For each setting, we re-ran the minimax search under $\lambda_0^\star$ to obtain a new minimax total sample size N and corresponding interim sample size $\tilde{N}$, together with the empirical operating characteristics $(\alpha_E, \text{Power}_E, \text{PET}_0, \text{PET}_1)$. The proportional change in minimax sample size is summarized by $\Delta_N = (N^*_{\text{mm}} - N_{\text{mm}})/(N_{\text{mm}})$, where N_{mm} denotes the minimax total sample size reported in Tables 1–3 under the nominal λ_0. The empirical power is slightly loose in a few rows because a coarser grid was used in searching over candidate total sample sizes.

As stated in Section 4.5, we do not keep the alternative hypothesis fixed while changing λ_0. Because uncertainty in λ_0 represents uncertainty in the baseline survival of the study population and thus affects both arms. Moreover, in both PH and NPH settings the alternative is specified relative to the control survival, so modifying λ_0 naturally induces a corresponding change in the experimental-arm hazard. This preserves the practical interpretation that the treatment effect is defined relative to the control arm rather than as an absolute experimental survival curve only.

Results in table S2 illustrate that re-optimizing the minimax design when λ_0 is misspecified can lead to either inflation or deflation of the required N in different scenarios. Under PH, a shift in λ_0 changes the amount of information available within the fixed restriction time and the censoring pattern. Therefore, a different (possibly larger) total sample size may be required to attain the target operating characteristics under the shifted null hypothesis. Under NPH settings, the behavior can be less monotone because changing λ_0 can alter both the RMST effect size accumulated over $[0, \tau]$ and the variance of the RMST estimators under censoring. These changes can interact differently across early- and late-difference alternatives. Thus, this robustness discussion based on required sample size is not directly analogous to robustness assessments for binary endpoints in Litwin et al.[2].

Table S2: Robustness: Minimax two-stage design when $\lambda_0^\star = 0.9\lambda_0$

α	**Power**	Δ	τ	**Method**	α_E	Power_E	N^*_{mm}	N_{mm}	Δ_N
**Proportional Hazards**									
0.05	0.8	1/1.5	2.5	Sim-RMST	0.0492	0.8118	312	176	0.7727
				Scu-RMST	0.0498	0.8246	150	128	0.1719
		1/1.7	2.5	Sim-RMST	0.0490	0.8002	138	104	0.3269
				Scu-RMST	0.0498	0.8138	106	80	0.3250
**Early Difference**									
0.05	0.8	0.5	2	Sim-RMST	0.0498	0.8174	94	88	0.0682
				Scu-RMST	0.0492	0.8323	64	70	-0.0857
		0.4	1.5	Sim-RMST	0.0496	0.8058	60	50	0.2000
				Scu-RMST	0.0492	0.8032	44	40	0.1000
**Late Difference**									
0.05	0.8	0.45	3	Sim-RMST	0.0493	0.8001	220	242	-0.0909
				Scu-RMST	0.0492	0.8032	170	208	-0.1827
		0.4	2.5	Sim-RMST	0.0490	0.8246	198	220	-0.1000
				Scu-RMST	0.0492	0.8032	160	194	-0.1753

References

[1] Huang B, Kuan PF. Comparison of the restricted mean survival time with the hazard ratio in superiority trials with a time-to-event end point. *Pharmaceutical Statistics.* 2018;17(3):202–213.

[2] Litwin S, Basickes S, Ross EA. Two-sample binary phase 2 trials with low type I error and low sample size. *Statistics in Medicine.* 2017;36(9):1383–1394.
